# A heterozygous deletion and inversion at the NHEJ1‑IHH locus associated with shank length in Yunlong short-leg chicken

**DOI:** 10.1186/s12864-026-12943-0

**Published:** 2026-05-18

**Authors:** Kuowei Xu, Kai Zhang, Jinyan Li, Yuxin Huang, Junlan Gou, Jielong Zhou, Yu Wang, Fenfen Chen, Ping Xiao

**Affiliations:** 1 College of Biological Science and Food Engineering, Southwest Forestry University, Kunming, Yunnan 650224 China; 2 Yunlong Short-Leg Chicken Conservation Farm of Dali Jimingjiang Breeding Chicken Co., Ltd., Dali Yunnan, 672799 China; 3 College of Animal Sciences and Technology, Northwest A & F University, Yangling, Shaanxi 712100 China

**Keywords:** Yunlong short-leg chicken, Whole-genome resequencing, Shank length, *IHH*, *NHEJ1*

## Abstract

**Background:**

The Yunlong Short-Leg chicken is a local dual-purpose breed in the Dali Bai Autonomous Prefecture of Yunnan Province, China, characterized phenotypically by notably shortened shank and a compact body stature. Nevertheless, the genetic mechanisms responsible for its short-legged phenotype have yet to be elucidated.

**Results:**

In this study, Yunlong Short-Leg chickens with different shank lengths—Long-legged and Short-legged phenotypes—were selected as experimental subjects (*n* = 60, 30 males and 30 females). We performed genome-wide analyses of single nucleotide polymorphisms (SNPs) and insertion/deletion variants (InDels), followed by genome-wide association studies (GWAS) correlating these variants with body weight and morphometric traits. Population genetic analysis indicated no significant differences in genetic background between the Long‑legged and Short‑legged groups, confirming they belong to the same population. GWAS identified shank length as the only trait significantly associated with eight genes on chromosome 7, including *IHH*, *NHEJ1*, and *MNR2*. A comparison of variants on chromosome 7 revealed that the Long‑legged samples exhibited no structural changes, whereas Short-legged samples were categorized as creeper phenotype, exhibiting two non-contiguous deletions and an inversion on chromosome 7: a 13,480 bp deletion (positions 22,287,390–22,300,869 bp) and an 11,559 bp deletion (positions 22,301,448–22,313,006 bp), totaling 25,039 bp, as well as a 578 bp inversion (positions 22,300,870–22,301,447 bp). Lethal samples carried a homozygous deletion and the same inversion in this region on chromosome 7. Multiplex PCR with different primers was performed to genotype shank-length phenotypes based on structural genomic variants. Finally, qRT‑PCR analysis confirmed that the relative mRNA expression levels of *IHH* and *NHEJ1* correlated with corresponding locus variation and shank-length phenotypes in Yunlong Short-Leg chicken.

**Conclusions:**

These findings provide insight into the genetic basis of the short‑leg phenotype in Yunlong Short-Leg chicken and offer theoretical support for genetic resource conservation and future breeding programs.

**Supplementary Information:**

The online version contains supplementary material available at 10.1186/s12864-026-12943-0.

## Background

The body size and stature of chickens are influenced by multiple factors, and the genetic basis of body conformation varies across different breeds [1]. Short-legged chickens are characterized by a relatively smaller body size and notably shorter shank length. Research on this phenotype dates back nearly a century [2]. Identification of the short-legged phenotype primarily relies on traits such as body weight, height, and shank length [3, 4]. Short-legged chickens are also referred to as bantam, creeping, or dwarf chickens [5], making it difficult to distinguish them from naturally small-bodied chicken breeds [6]. The genetic mechanisms underlying these phenotypes differ among breeds. 

“Short-legged” is a general descriptive term referring to chickens with markedly shortened shank length and reduced body size, which can arise from different genetic mechanisms across breeds. “Bantam” refers to a form of proportional dwarfism characterized by overall small body size. Recent genomic studies have demonstrated that the bantam phenotype in Dutch chickens is heterogeneous and associated with variants in genes such as *HMGA2* and *PRDM16*, which are distinct from the creeper locus [7]. “Dwarf” is a broad term encompassing multiple forms of growth reduction, including sex-linked dwarfism (dw) [3] and autosomal dwarfism (adw) associated with *TMEM263* mutations [8]. “Creeper” (Cp) is a specific genetic syndrome characterized by autosomal dominant chondrodystrophy in heterozygotes (Cp/+) and embryonic lethality in homozygotes (Cp/Cp) [9]. The creeper phenotype has been shown to be caused by deletions of *IHH* alone in Xingyi bantam chickens [10] or combined deletions of *IHH* and *NHEJ1* in Japanese bantam chickens [11]. As a rare short-legged chicken breed in China, the Yunlong Short-Leg chicken is particularly known for its reduced shank length and compact body size [12]. Although the genetic basis of corresponding phenotypes in chickens has been elucidated, the formation mechanism of the short-legged phenotype in Yunlong Short-Leg chickens remains unreported.

To identify functional genes associated with the shank length of Yunlong Short-Leg chickens, this study conducted whole-genome resequencing on long-legged and short-legged populations, followed by GWAS based on different types of molecular markers. The goal was to detect genomic variants significantly associated with shank length and to characterize the structural variations at the candidate locus. This study aims to reveal the genetic basis of the short-legged phenotype in Yunlong Short-Leg chickens and to provide theoretical support for genetic resource conservation and future breeding programs.

## Materials and methods

### Experimental design and sample collection

All Yunlong Short-Leg chickens were sampled from the Yunlong Short-Leg Chicken Conservation Farm, affiliated with Jimingjiang Poultry Breeding Co., Ltd., located in Dali Bai Autonomous Prefecture, China. All birds were raised under uniform feeding and immunization conditions. A total of 60 individuals at 340 days of age were selected for this study, including 28 Long-legged and 32 Short-legged individuals, with equal representation of males and females in each phenotype. Blood samples were collected from the underwing vein and stored in EDTA anticoagulant tubes, then immediately placed in a low-temperature freezer for genomic DNA extraction.

Fertilized eggs produced by crossing short-legged males and short-legged females were used to obtain lethal-type samples. The incubation experiment was conducted in the hatchery of the College of Biological Science and Food Engineering, Southwest Forestry University. Candling of eggs was performed daily during the early incubation stage, and embryo tissues from individuals that died on day 4 were collected and stored at -80 °C for DNA and RNA extraction. On the first day of hatching, the chicks were euthanized by carotid artery bloodletting. Blood and metatarsal growth plate tissues were collected in 2 mL enzyme-free centrifuge tubes, immediately immersed in liquid nitrogen, and subsequently stored at − 80 °C for DNA and RNA extraction, respectively.

### Genomic data acquisition of other chicken breeds

Whole-genome resequencing data of Red Jungle fowl (Gallus gallus, *n* = 10), Nixi chickens (*n* = 11), and Dulong chickens (*n* = 10) were downloaded from the NCBI database (accession numbers: PRJNA782225, PRJNA782225, and PRJNA559932, respectively), while data from Ninglang Plateau chickens (*n* = 20) were obtained from the National Genomics Data Center (accession: PRJCA026315). Note that Red Junglefowl and Nixi chickens share the same BioProject accession number (PRJNA782225) as they originate from the same sequencing project.

### Phenotypic recording

Body weight and body size traits were measured for 60 Yunlong Short-Leg chickens according to the national standard NY/T 823—2020, “Terminology and Measurement for Poultry Productive Performance” [13]. Measured body size traits included body slant length, keel length, shank length, shank circumference, chest depth, chest width, hip width, and breast angle.

### DNA and RNA extraction

Genomic DNA from blood samples was extracted using a blood genomic DNA purification kit, while DNA from embryo tissues was extracted using a standard genomic DNA kit. Both kits were obtained from Beijing TransGen Biotechnology Co., Ltd. DNA integrity was verified using 1% agarose gel electrophoresis. Qualified DNA samples were sent to Wuhan Yinzhi Gene Technology Co., Ltd. for library construction and paired-end sequencing. Total RNA was extracted using the RNAiso Plus kit (Cat No. 9108) following the manufacturer’s instructions. RNA integrity was assessed via 1% agarose gel electrophoresis, and concentration and purity were measured using a NanoVue Plus microvolume spectrophotometer. Qualified RNA was reverse-transcribed to cDNA and the rest was stored at -80 °C, while cDNA was stored at -20 °C for further use.

### Variant calling and genotyping

DNA sequencing was performed on the MGISEQ-T7 platform with 150 bp paired-end reads. Raw reads were quality-filtered using Fastp v0.23.2 [14] to remove adapters, reads with > 3% ambiguous bases, reads shorter than 30 bp, and reads with > 40% low-quality bases (Q < 15). Clean reads were aligned to the chicken reference genome (GRCg6a, GCA_000002315.6) using BWA v0.7.17 [15]. Alignments were sorted using “sort” function from samtools v1.10.2 [16], and PCR duplicates were removed using Picard v2.1. SNPs and InDels were called and genotyped using GATK v4.2.0 [17] with the “HaplotypeCaller,” “GenotypeGVCFs,” and “SelectVariants” modules. Variants were filtered using GATK’s “VariantFiltration” module with the following thresholds: QD < 2.0, MQ < 40.0, FS > 60.0, SOR > 3.0, MQRankSum < − 12.5, and ReadPosRankSum < − 8.0. Population-level SNPs were filtered with a minor allele frequency (MAF) > 0.05 and missing rate < 0.1. Variant annotation and chromosomal distribution analysis were conducted using ANNOVAR [18].

### Population genetic structure

Principal component analysis was performed using PLINK v1.90 [19], and a neighbor-joining (NJ) tree was constructed using PHYLIP v3.696 [20]. The phylogenetic tree was visualized using tvBOT [21]. In order to eliminate SNPs in linkage disequilibrium (LD), LD pruning was conducted using the PLINK option “–indep-pairwise 50 5 0.2”. ADMIXTURE (v1.3.0) [22] was employed to perform population structure analysis, assuming the number of ancestral populations (subpopulations) ranged from K = 1 to K = 4. Cross-validation was conducted to estimate individual ancestry proportions and admixture levels, and the cross-validation error at different K values was calculated to determine the optimal K. The PCA and population structure analysis results were visualized using R (v4.5.1).

### Genome-wide association analysis for multiple body size traits

A mixed linear model implemented in GEMMA [23] (v0.98.5) was used to perform genome-wide association analysis (GWAS) of body weight and body size traits in Yunlong Short-Leg chickens. The following statistical model was applied: *y = Wα + Xβ + u + e*, where *y* is the vector of phenotypic values; *W* is an n × p matrix of covariates (fixed effects), including the sex as well as top three principal components; *α* is a vector of the corresponding coefficients including the intercept; *X* is the vector of marker genotypes; *β* represents the effect size of the marker and is an estimate of its additive effect; *u* denotes the vector of random polygenic effects; and e is the residual error vector. The Bonferroni correction method was applied to adjust for multiple testing. A SNP was considered significantly associated with the trait if its *P*-value was less than 0.01/N (where N is the total number of SNPs retained after quality control), and suggestively associated if the *P*-value was less than 0.05/N. The genome-wide significance threshold was set at 0.05/N. Manhattan plots and Q-Q plots were generated using the qqman and CMplot packages in R. Significant associated variants were annotated using ANNOVAR [18].

### Primer design and sequencing validation

PCR primers were designed using Primer Premier 6.0. For qPCR, each primer pair was designed to span at least one intron to avoid genomic DNA amplification. During the primer design process, the formation of primer dimers, hairpin structures, and nonspecific amplification between primer pairs was optimized. Primer pairs were selected to have similar melting temperatures and GC content. Primer specificity was verified using the UCSC In-Silico PCR tool (https://genome.ucsc.edu/cgi-bin/hgPcr).

All primers were synthesized by Sangon Biotech Co., Ltd. (Shanghai, China).

The primer pair WT-F(5’-GGATGCCGTAGGATGGAT-3’) and Common-R(5’-GCTGGTTAATCAGGCTGAG-3’) amplified a 635 bp product in wild-type chickens without mutations. The MT-F(5’-TCGCTGAGACAACAATGAG-3’) and Common-R(5’-GCTGGTTAATCAGGCTGAG-3’) primer pair amplified a 408 bp fragment in individuals with deletions and inversions. Besides multiplex PCR design, the Del-F(5’-AGAGTGTGTATGTGCTTCC-3’) and Del-R(5’-CTCGTTAAGCTGACACCTC-3’) primers were used for conventional PCR to verify the deletion-inversion region, yielding a 1,130 bp product in mutant genotypes.

The multiplex PCR reaction system used in this study contained a total volume of 25 µL: 12.5 µL of 2× PCR Mix, 0.4 µL of primer WT-F (10 µmol/L), 0.4 µL of primer MT-F (10 µmol/L), 0.8 µL of primer Common-R (10 µmol/L), 2 µL of template DNA (50–100 ng/µL), and 8.9 µL of ultrapure water. The thermal cycling conditions were as follows: initial denaturation at 95 °C for 5 min; 35 cycles of denaturation at 95 °C for 30 s, annealing at 62 °C for 30 s, and extension at 72 °C for 35 s; final extension at 72 °C for 10 min; and storage at 4 °C. The multiplex PCR products were analyzed by 2% agarose gel electrophoresis at 120 V and 100 mA for 30 min. After electrophoresis, the gels were visualized using a gel documentation system. PCR products were sent to Sangon Biotech Co., Ltd. (Shanghai, China) for T-vector sequencing. Sequence alignment and validation were performed using DNAman v9.0 to confirm the consistency between the amplified and expected sequences.

### Quantitative RT-PCR analysis of candidate gene expression

Total RNA extracted from individuals with different phenotypes was reverse transcribed into cDNA using a commercial reverse transcription kit designed for quantitative PCR, according to the manufacturer’s instructions. The resulting cDNA was used as a template for qRT-PCR to evaluate the relative mRNA expression levels of the *IHH* and *NHEJ1* genes. The *β-actin* gene was used as the internal reference. Samples from each phenotype (long-legged, short-legged and lethal types) were analyzed with three biological replicates and three technical replicates.

The total volume of the qRT-PCR reaction was 15 µL, comprising 1.5 µL of template cDNA, 7.5 µL of 2× TB Green^®^ Premix Ex Taq™ II (Tli RNaseH Plus), 0.6 µL each of forward and reverse primers, and 4.8 µL of ddH₂O. The amplification program was as follows: initial denaturation at 95 °C for 30 s; 39 cycles of denaturation at 95 °C for 5 s, annealing at 60 °C for 30 s, and extension at 72 °C for 30 s. All primers were synthesized by Sangon Biotech Co., Ltd. (Shanghai, China), and the primer sequences are listed in Supplementary Table 1.

The relative gene expression levels were calculated using the 2^^−ΔΔCt^ method, and data visualization was performed using GraphPad Prism 8.0.

### Data statistical analysis

Body weight and body size data were organized and analyzed using Excel. One-way analysis of variance (ANOVA) and independent sample t-tests were performed using SPSS 22.0 software (SPSS Science, Chicago, IL, USA). Data are presented as mean ± standard error of the mean (SEM). * indicates a statistically significant difference (*P* < 0.05), and ** indicates a highly significant difference (*P* < 0.01). Means sharing the same letter are not significantly different (*P* > 0.05), whereas means with different letters differ significantly (*P* < 0.05). Expression levels of candidate genes were visualized using GraphPad Prism 8.0, and genomic data were visualized using R software (v4.5.1).

## Results

### Significant differences in body size traits between long-legged and short-legged Yunlong short-leg chickens

**Fig. 1:**
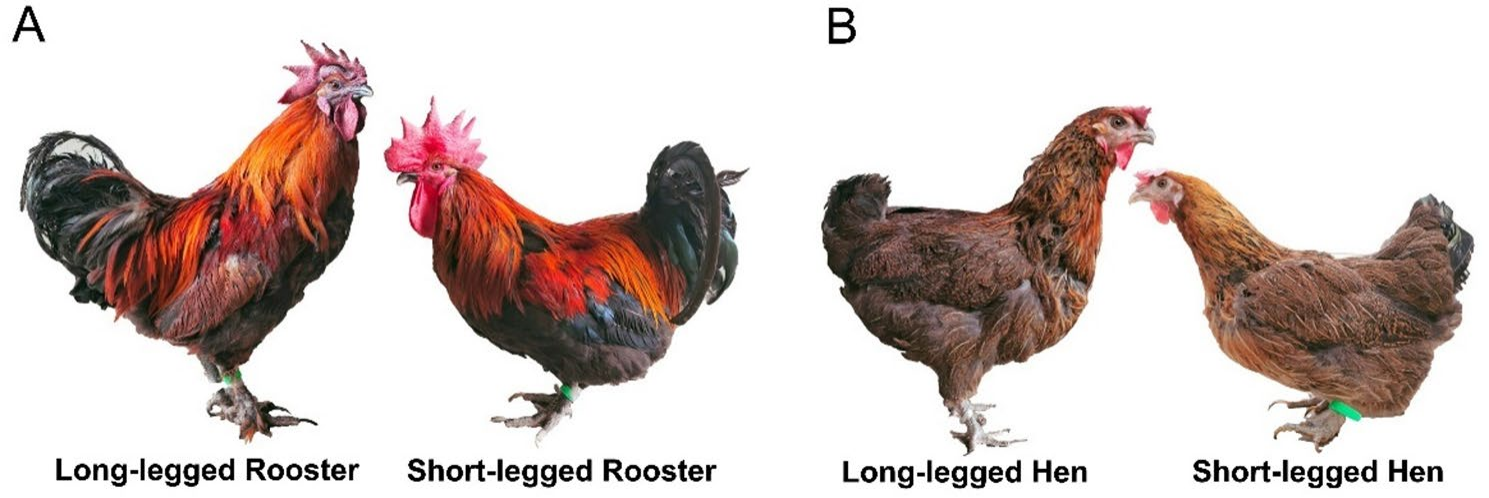
Appearance characteristics of Yunlong short-Leg chicken. **A **Side view of long-legged and short-legged rooster. **B **Side view of long-legged and short-legged hen

At 340 days of age, the phenotypic characteristics of Yunlong Short-Leg chickens were recorded, as shown in Fig. 1. Short-legged Yunlong Short-Leg chickens, both male and female, were characterized by relatively shorter shank lengths and smaller body size. In contrast, Long-legged individuals displayed larger overall body sizes (Fig. 1A and B). Body weight and body size traits were measured in all individuals (Supplementary Table 2). The results for different phenotypes were presented in Table 1. The average shank length of Long-legged males was 95.53 mm, compared to 70.65 mm in Short-legged males. Similarly, the average shank length of Long-legged females was 80.38 mm, whereas that of Short-legged females was 60.40 mm. Additionally, within the same sex, both body slant length and shank length were significantly greater in Long-legged individuals than in the Short-legged counterparts (P < 0.05).

**Table 1:** Mean and standard deviation (SD) of the morphometric traits of Yunlong short-Leg chicken

Type	Long-legged Rooster (*n* = 14)	Short-legged Rooster (*n* = 16)	Long-legged Hen (*n* = 14)	Short-legged Hen (*n* = 16)
Body weight(kg)	2.38 ± 0.39^a^	2.12 ± 0.30^b^	1.89 ± 0.31^b^	1.71 ± 0.27^c^
Body slant length(cm)	27.11 ± 1.67^a^	21.56 ± 2.28^c^	23.10 ± 1.66^b^	20.20 ± 1.20^d^
Keel length(cm)	11.26 ± 0.86^a^	10.76 ± 0.62^a^	9.79 ± 0.84^b^	9.48 ± 0.61^b^
Shank length(mm)	95.53 ± 7.37^a^	70.65 ± 5.10^c^	80.38 ± 5.01^b^	60.4 ± 4.48^d^
Shank circumference(cm)	4.74 ± 0.51^a^	4.54 ± 0.59^a^	3.80 ± 0.50^b^	3.84 ± 0.60^b^
Chest depth(mm)	115.35 ± 11.03^a^	110.72 ± 13.97^ab^	105.44 ± 8.04^b^	95.54 ± 9.96^c^
Chest width(mm)	92.46 ± 8.47^a^	95.61 ± 8.42^a^	78.12 ± 6.90^b^	72.14 ± 8.96^b^
Hip width(mm)	60.38 ± 6.29^b^	70.27 ± 5.68^a^	55.54 ± 3.98^c^	55.92 ± 4.14^c^
Chest angle(°)	115.03 ± 8.26^a^	99.96 ± 7.31^b^	95.11 ± 10.25^b^	100.04 ± 9.46^b^

Different letters in the same row indicate significant differences (*P* < 0.05), while the same letters indicate no significant differences (*P* > 0.05)

### Genetic variation and annotation in Yunlong short-Leg chicken

Quality control metrics for whole-genome sequencing were provided in Supplementary Table 3. Low-quality variants were filtered using GATK, resulting in a total of 21,116,000 SNPs and 2,931,696 InDels identified across all Yunlong Short-Leg chicken samples. These variants were annotated using ANNOVAR, and the results were summarized in Supplementary Table 4. Most SNPs were located within intronic regions (49.07%), followed by intergenic regions (32.77%), with a smaller proportion located in exonic regions (1.67%). The remaining 16.48% were distributed across other genomic regions. A total of 217,963 synonymous SNPs and 130,159 nonsynonymous SNPs were identified in exonic regions. InDel annotation revealed that 49.47% were located in introns, 33.57% in intergenic regions, and only 0.42% in exons. Among exonic InDels, 7,424 were annotated as synonymous and 4,206 as nonsynonymous. The remaining 16.54% belonged to other genomic categories.

### No genetic background differences between long-legged and short-legged Yunlong short-leg chickens

**Fig. 2:**
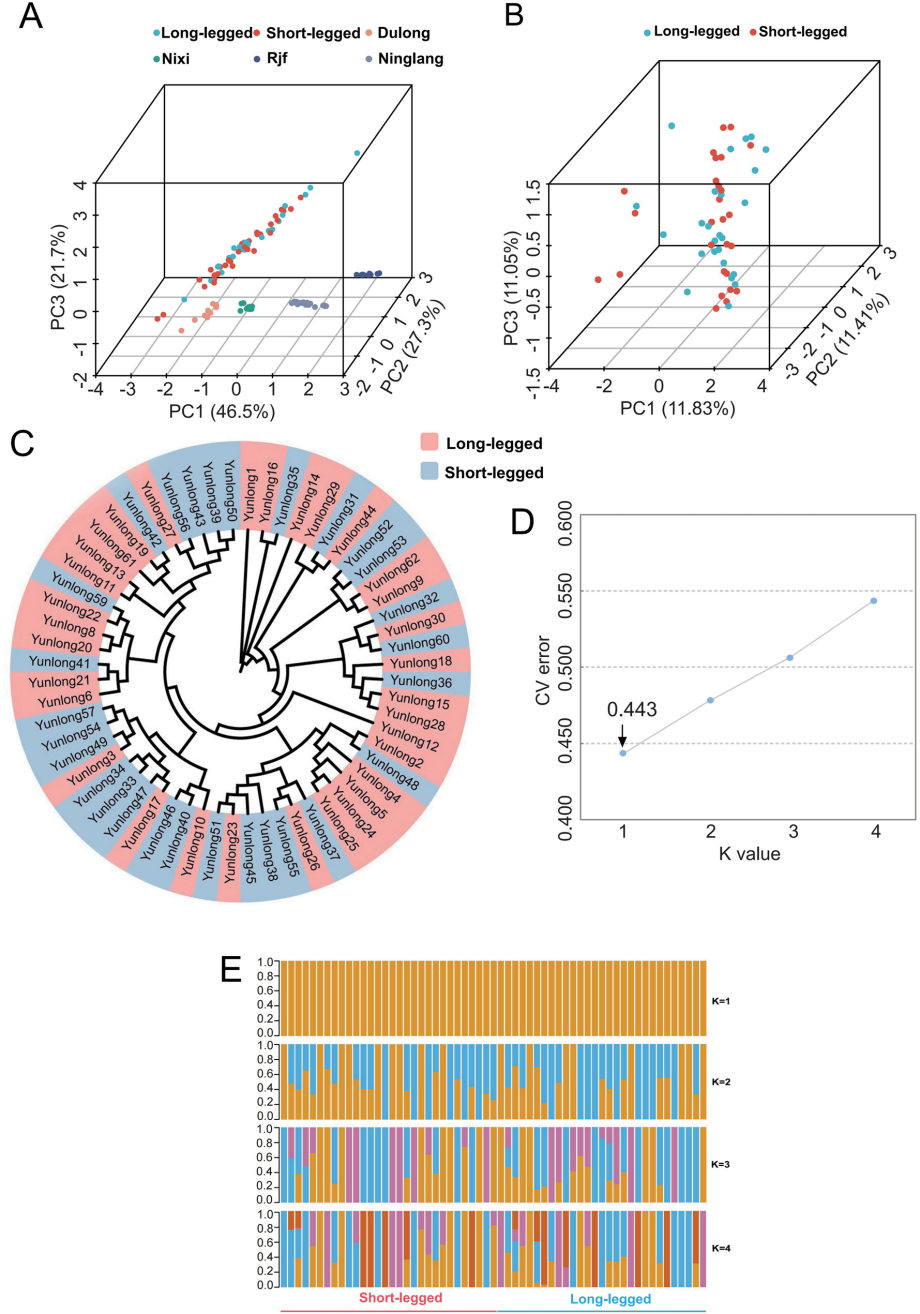
Genetic structure of Yunlong short-leg chicken population. **A** Principal component analysis of different local chicken breeds in Yunnan. **B** Principal component analysis of Yunlong short-leg chickens with different phenotypes. **C** Phylogenetic tree. **D** Cross-validation error rate. **E** Population structure diagram

Population structure analysis was conducted on the Yunlong Short-Leg chicken population to assess genetic differentiation between Long-legged and Short-legged individuals. Principal component analysis (PCA), neighbor-joining (NJ) tree, and ADMIXTURE were employed. The PCA results revealed that Yunlong Short-Leg chickens were genetically distinct from other native chicken breeds in Yunnan Province (Fig. 2A), whereas no genetic differentiation was observed between the Long-legged and Short-legged subpopulations (Fig. 2B). To further investigate the genetic relationship between the two leg-type groups, a phylogenetic tree was constructed using the neighbor-joining algorithm. The results showed that Long-legged and Short-legged Yunlong Short-Leg chickens clustered together (Fig. 2C). Ancestry inference based on ADMIXTURE indicated that the optimal number of ancestral populations occurred at K = 1 (Fig. 2D), and no differentiation in genetic background was observed between the two groups at K ≥ 2 (Fig. 2E). Overall, PCA, NJ tree, and population structure analyses confirmed that Long-legged and Short-legged Yunlong Short-Leg chickens share the same genetic background and belong to a single population.

### Genome-wide association study (GWAS) identifies candidate genes on chromosome 7 associated with shank length

**Fig. 3:**
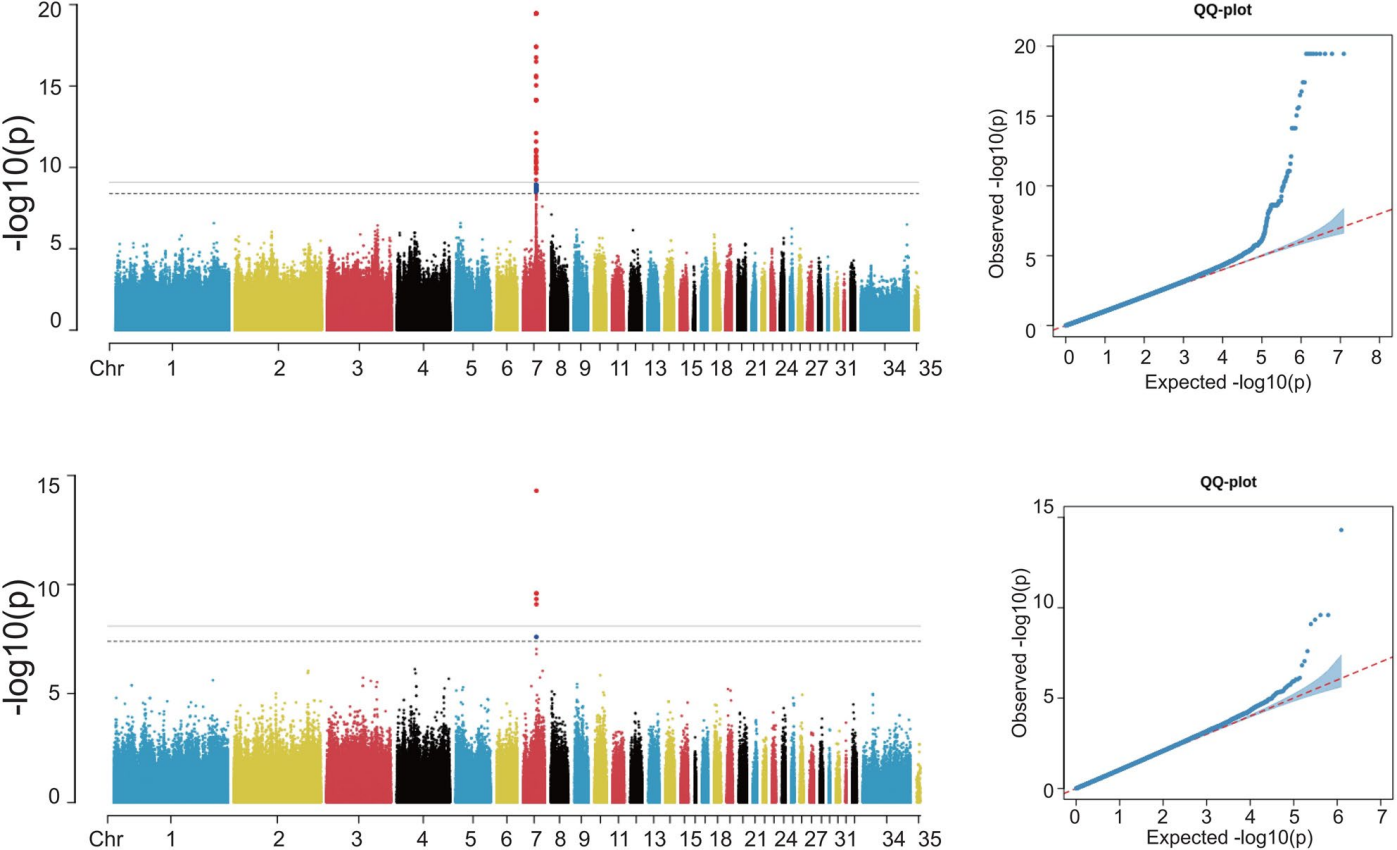
Results of GWAS association analysis with shank length traits. **A** GWAS based on whole genome SNPs and shank length trait. **B** GWAS based on whole genome InDels and shank length trait

A genome-wide association study (GWAS) was performed using the mixed linear model implemented in GEMMA to identify loci associated with body size traits in Yunlong Short-Leg chickens. The genome-wide significance threshold was set at 0.05/N, where N represents the number of variants remaining after PLINK filtering. The results indicated that only shank length was associated with genome-wide significant SNP and InDel signals (Fig. 3A and B), while no other body size traits showed significant associations. A total of 79 SNPs and 6 InDels reached genome-wide significance for shank length. Linkage disequilibrium (LD) analysis of SNPs near the *IHH* gene revealed strong LD among several significantly associated SNPs (Supplementary Fig. 1). Annotation of all significant variants using ANNOVAR was presented in Supplementary Table 5. All genome-wide significant SNPs were located on chromosome 7, including 7 in exonic regions, 5 in intergenic regions, 50 in intronic regions, and 17 in other genomic regions. The associated genes included *DNPEP*, *FAM134A*, *CNPPD1*, *SLC23A3*, *NHEJ1*, *IHH*, *MNR2*, and *FEV*. All significantly associated InDels were also located on chromosome 7, with 2 in intergenic regions and 4 in intronic regions. These were annotated to *FAM134A*, *CNPPD1*, *IHH*, and *MNR2* genes.

### Genomic characteristics of the lethal type in Yunlong short-leg chickens

Based on the genome-wide association signals of SNPs and InDels for shank length, the *IHH* gene was identified as a candidate gene. This finding suggests that the Yunlong Short-Leg chicken population may carry the Creeper gene, and that the shank length phenotype is associated with *IHH* gene mutations. *IHH* is recognized as the Creeper gene, whose heterozygous deletion causes the Creeper phenotype, while homozygous deletion leads to early embryonic lethality. The lethal type embryos typically exhibit underdeveloped yolk sac vasculature, delayed brain growth, arrested heartbeat, abnormal embryonic morphology, and a circular vascular distribution surrounding the embryo [10, 11]. To obtain lethal-type embryo samples, fertilized eggs were produced by mating Short-legged males and females, followed by artificial incubation with daily monitoring of embryonic development. Morphological dissection of embryonated eggs at embryonic day 4 (E4) is shown in Supplementary Fig. 2. Among the lethal-type embryos observed at E4, six representative individuals were selected for DNA extraction and subjected to whole-genome resequencing. The alignment files were visualized using IGV software (Supplementary Fig. 3).

Structural variant analysis revealed inverted sequences at chromosome 7 positions 22,287,389–22,301,447 bp and 22,300,870–22,313,007 bp, corresponding to paired reads shown in green and blue in Supplementary Fig. 3. The deleted segments span from 22,287,390–22,300,869 bp and from 22,301,448–22,313,006 bp, totaling 25,039 bp, while the inversion was confined to 22,300,870–22,301,447 bp, with a length of 578 bp. These two deleted regions span from intron 4 to exon 7 of the *NHEJ1* gene and the entire *IHH* gene. The inverted segment is located in the intergenic region between *IHH* and *NHEJ1*.

### Genotyping of shank-length mutations in Yunlong short-leg chickens using multiplex PCR

**Fig. 4:**
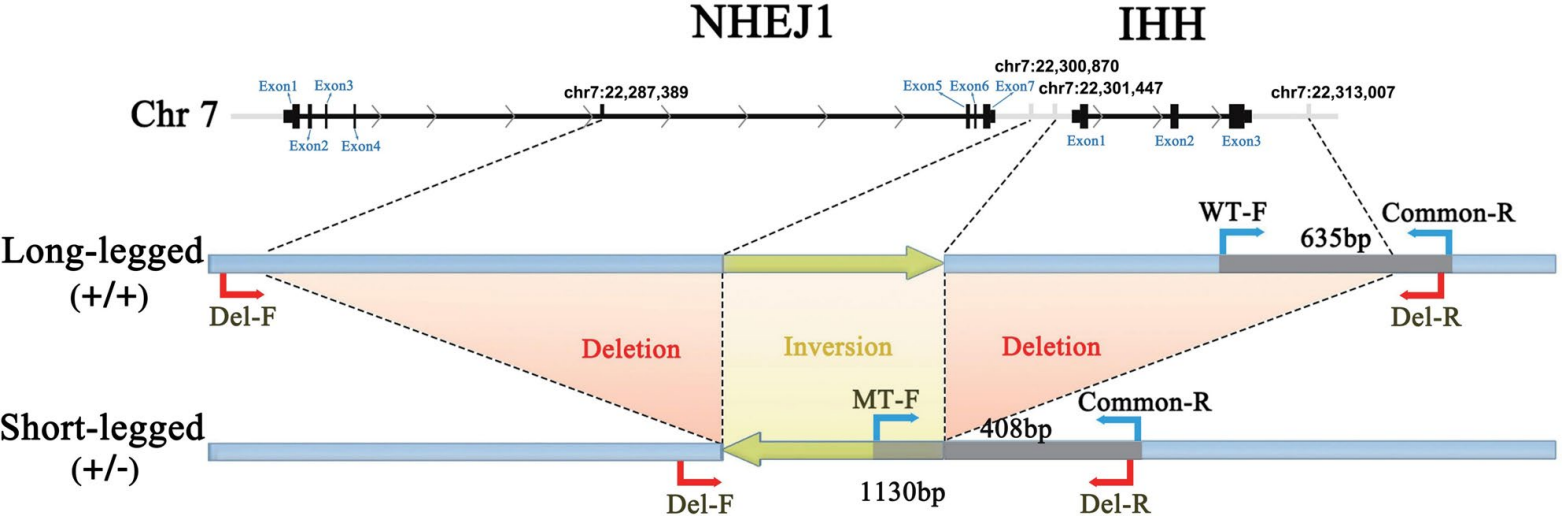
Schematic diagram of multiplex PCR primer design

To enable rapid and accurate differentiation of genomic variations in Yunlong Short-Leg chickens, we designed a multiplex PCR assay targeting the different mutation types observed in Long-legged, Short-legged, and lethal individuals. Primer design is shown in Fig. 4. The primer pair WT-F and Common-R amplified a 635 bp product in wild-type chickens without mutations. The MT-F and Common-R primer pair amplified a 408 bp fragment in individuals with deletions and inversions. Besides multiplex PCR design, the Del-F and Del-R primers were used for conventional PCR to verify the deletion-inversion region, yielding a 1,130 bp product in mutant genotypes.

**Fig. 5:**
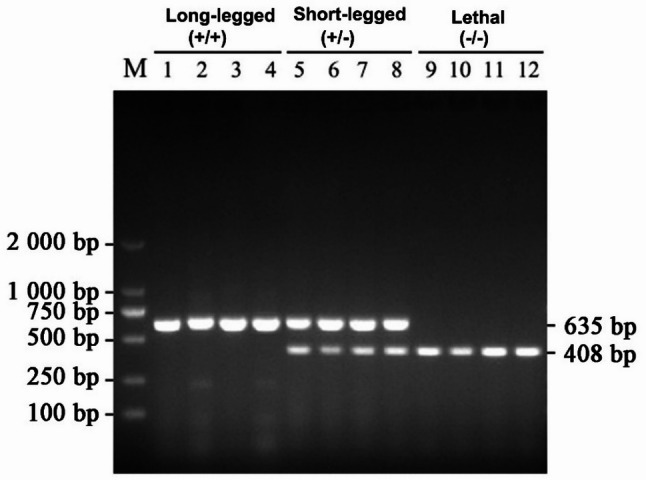
Genotyping results of Yunlong short-leg chickens with different phenotypes

The results of the multiplex PCR genotyping are shown in Fig. 5. Individuals 1–4 were Long-legged and showed only the 635 bp wild-type band. Individuals 5–8 were Short-legged and exhibited both 635 bp and 408 bp bands, indicating heterozygosity. Individuals 9–12 were lethal-type and showed only the 408 bp mutant band, consistent with homozygous mutation. Therefore, the number and size of amplified bands in the multiplex PCR assay can reliably distinguish shank-length genotypes in Yunlong Short-Leg chickens.

The Del-F and Del-R primers were also used to amplify the deleted-inverted region by conventional PCR in different genotypes. As shown in Supplementary Fig. 4, individuals 1–4 (Long-legged) had no amplification product, indicating the absence of mutations in *IHH* and *NHEJ1*. Individuals 5–8 (Short-legged) showed a 1,130 bp band, consistent with heterozygous mutations. Individuals 9–12 (lethal-type) also showed a 1,130 bp band, consistent with homozygous mutations. PCR products from Short-legged and lethal individuals were cloned into T vectors and sequenced. The sequencing results are shown in Supplementary Fig. 5. The deleted regions were located at 22,287,390–22,300,869 bp (13,480 bp) and 22,301,448–22,313,006 bp (11,559 bp), while the 578 bp inverted segment was located at 22,300,870–22,301,447 bp, highlighted in yellow.

### Structural variation in chromosome 7 among different genotypes of Yunlong short-leg chickens

**Fig. 6:**
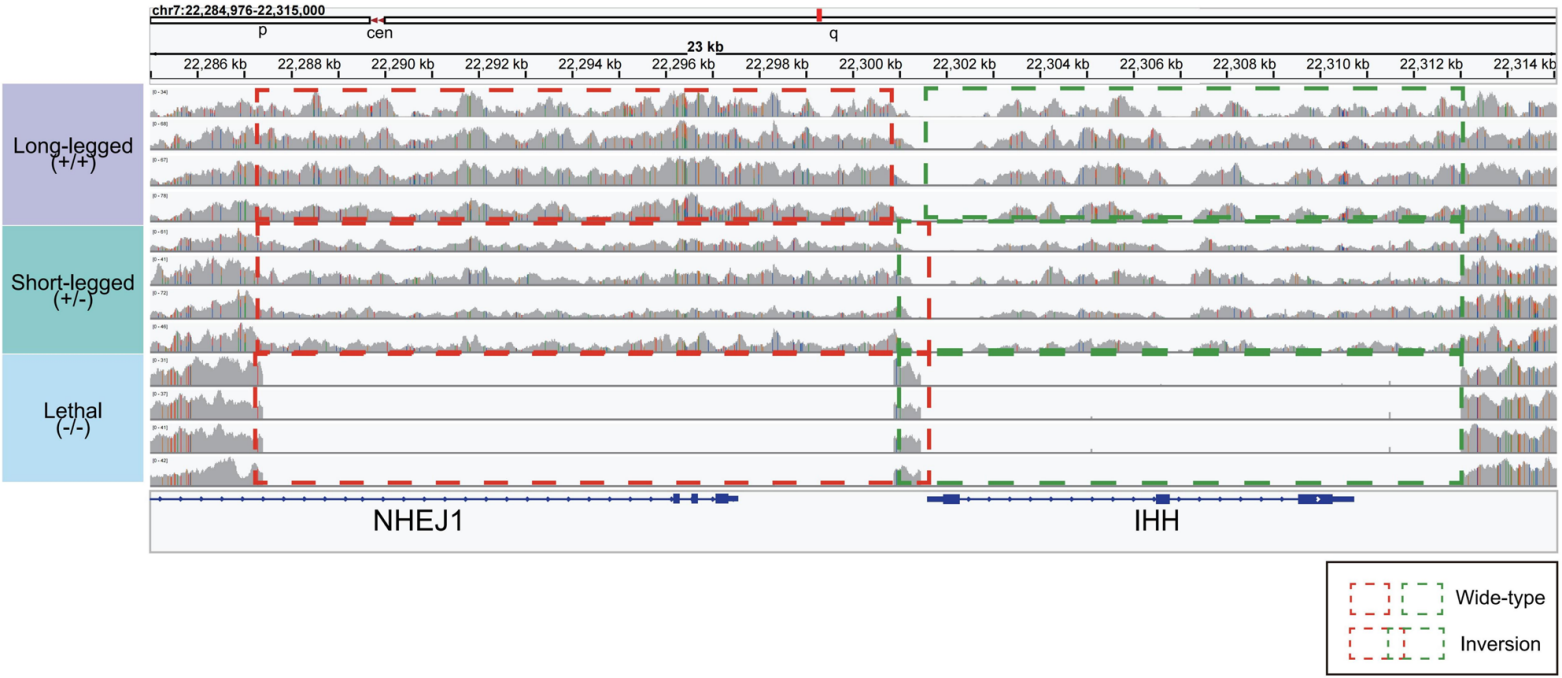
Comparison of variation among different genotypes of Yunlong short-leg chicken

Paired-end sequencing data from Long-legged, Short-legged, and lethal-type Yunlong Short-Leg chickens were analyzed in IGV to visualize candidate regions identified from GWAS. As shown in Fig. 6, lethal individuals exhibited deletions in chromosome 7 regions 22,287,390–22,300,869 bp and 22,301,448–22,313,006 bp, along with an inversion from 22,300,870–22,301,447 bp. The deleted segments encompass intron 4 to exon 7 of the *NHEJ1* gene and the entire *IHH* gene. The inversion was located in the intergenic region between *IHH* and *NHEJ1*. These deleted regions exhibited low sequencing coverage in Short-legged individuals, but no structural variation was observed in Long-legged individuals. Thus, individuals with homozygous mutations (−/−) in this region exhibited lethal phenotypes; those with heterozygous mutations (+/−) displayed the Short-legged phenotype; and individuals without mutations (+/+) exhibited the Long-legged (wild-type) phenotype.

### Analysis of mRNA expression levels of candidate genes in Yunlong short-leg chickens with different genotypes

**Fig. 7:**
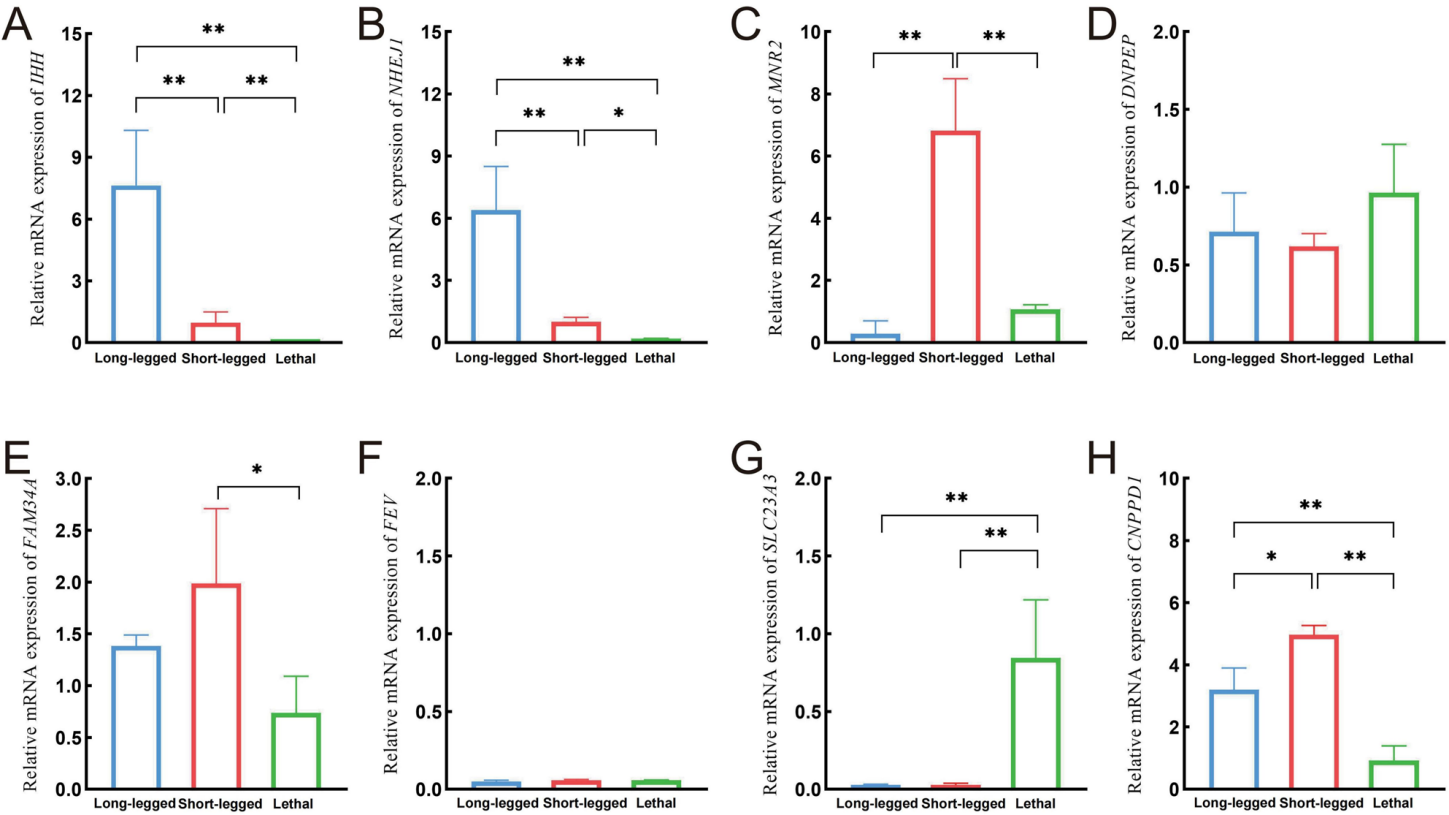
Relative mRNA expression levels of *IHH* (**A**), *NHEJ1* (**B**), *MNR2* (**C**), *DNPEP* (**D**), *FAM34A* (**E**), *FEV* (**F**), *SLC23A3* (**G**), and *CNPPD1* (**H**) in individuals with different genotypes

GWAS based on SNPs and InDels for shank length identified candidate genes including *DNPEP*, *FAM134A*, *CNPPD1*, *SLC23A3*, *NHEJ1*, *IHH*, *MNR2*, and *FEV*. To investigate the expression patterns of these genes between Long-legged and Short-legged individuals at Day 1 post-hatch, and the lethal-type samples at E4 are presented as supplementary observation, quantitative real-time PCR (qRT-PCR) was performed to assess the relative mRNA expression levels of shank-length-associated genes. As shown in Fig. 7, the relative expression levels of *IHH* and *NHEJ1* were significantly higher in Long-legged individuals compared to Short-legged individuals, and were completely absent in lethal-type individuals (Fig. 7A and B). These results were consistent with the observed genomic deletions in the corresponding regions of these genes among different genotypes.

## Discussion

The Short-legged trait in chickens has been extensively studied. Chickens exhibiting the Short-legged phenotype are mainly characterized by shortened limbs and reduced body size [9]. The identification of these phenotypes in chickens is primarily based on body weight, height, and shank length, which complicates the distinction between Short-legged, bantam, Creeper, and dwarf chickens [6]. Several genes, including Cp [24], td [25], dw [26], and adw [3], have been linked to these phenotypes in poultry. The Creeper phenotype is caused by the autosomal dominant mutation [24]. To date, the Creeper phenotype has been associated with different structural variants across breeds: a deletion of *IHH* alone in Xingyi short-legged chickens, and a combined deletion of *IHH* and *NHEJ1* in Japanese bantam chickens. Investigating the formation and regulatory mechanisms of Short-legged phenotypes across various Short-legged chicken breeds is essential for a deeper understanding of the complexity of this phenotype. In this study, genome-wide association analysis (GWAS) was employed to identify genomic variations linked to the Creeper phenotype in Yunlong Short-Leg chickens, and the expression levels of the *IHH* and *NHEJ1* genes were validated across distinct genotypes.

GWAS is widely utilized to identify genomic variants statistically associated with diseases or specific traits [27] and has been extensively applied in studies of complex traits in poultry [28, 29]. In this study, GWAS was conducted on shank length and other morphological traits of Yunlong Short-Leg chickens. Significant SNP and InDel signals were detected exclusively for shank length, while no significant loci were identified for other morphological traits. The following aspects may account for this observation.

First, the Cp mutation directly affects endochondral bone formation via *IHH* dysregulation, and the shank (tibiotarsus) is the most rapidly growing long bone in chickens [30]. Therefore, the primary phenotypic target of the *IHH* deletion is longitudinal bone growth, which is directly reflected in shank length. Second, other traits such as body weight, chest depth, and chest width may show differences secondary to the overall reduction in body size rather than being directly regulated by the IHH‑NHEJ1 locus. In other words, smaller body stature leads to proportional reductions in multiple body measurements, but these traits are polygenic [31, 32] and influenced by many other loci. Third, the statistical power of GWAS is highest for traits with a strong single-locus effect (like shank length). For traits with more complex or polygenic architecture, our sample size (*n* = 60) may be insufficient to detect genome-wide significant associations. Fourth, similar observations have been reported in other short-legged chicken breeds: Jin et al. [10] and Kinoshita et al. [11] also identified *IHH* deletions as the primary driver of shank shortening, with other body size traits showing less stringent association signals. All significant SNPs were annotated to chromosome 7 of the Yunlong Short-Leg chicken genome, with genes annotated at significant SNP loci including *DNPEP*, *FAM134A*, *CNPPD1*, *SLC23A3*, *NHEJ1*, *IHH*, *MNR2*, and *FEV*. The significant InDels were annotated to the *FAM134A*, *CNPPD1*, *IHH*, and *MNR2* genes. The candidate genes identified in this study differ from those identified in Dutch bantam and Italian local dwarf chicken breeds. Wu et al. [7] conducted GWAS on Dutch bantams and identified *HMGA2* and *PRDM16* as genes associated with the phenotype, both of which are involved in chicken body growth and development. Perini et al. [6] performed GWAS on three Italian local dwarf chicken breeds and identified *LEMD3* and *HMGA2* genes as associated with dwarfism. These findings suggest that different genes may determine these phenotypes in different Short-legged chicken breeds.

The *IHH* gene encodes a member of the Hedgehog protein family [33] and plays a critical role in regulating bone growth and development. It is essential for chondrocyte proliferation, differentiation, and osteogenesis [34]. Mutations in *IHH* have been implicated in human type A1 brachydactyly and femoral dysplasia, conditions characterized by limb shortening, reduced stature, and shortened digits [35–37]. Growth hormone therapy has proven effective in improving these conditions [38]. Knockout of *IHH* in mice results in shortened limbs and suppressed chondrocyte proliferation [39].

These findings underscore the crucial role of *IHH* in skeletal development and suggest that mutations in *IHH* may contribute to the Short-legged phenotype in Yunlong Short-Leg chickens. Furthermore, linkage disequilibrium analysis of SNPs near the candidate regions identified by GWAS revealed a strong linkage between the *IHH* gene and *MNR2*, the latter of which regulates rose comb formation in chickens [40]. SNPs near *IHH* and *MNR2* formed haplotype blocks, consistent with previous studies [24, 40, 41], indicating a tight linkage between the *IHH* gene, which controls the Creeper trait, and the *MNR2* gene, which regulates rose comb morphology. Classical genetic experiments and inheritance analyses have demonstrated that the Creeper trait is controlled by the semi-lethal dominant Cp allele [11]. Heterozygous individuals (Cp/+) exhibit the Creeper phenotype, wild-type homozygotes (+/+) show normal phenotypes, and homozygous mutants (Cp/Cp) exhibit lethality, typically occurring around embryonic day four. Jin et al. [10] were the first to identify a deletion in *IHH* as responsible for the Creeper trait in Xingyi Short-legged chickens. Kinoshita et al. [11] discovered that the Creeper phenotype in Japanese bantam chickens was due to a combined deletion of *IHH* and *NHEJ1*. In our study, genome sequencing revealed that the read depth across the *IHH* gene was markedly lower in Short-legged Yunlong Short-Leg chickens compared to Long-legged individuals (as visualized in IGV), suggesting a potential hemizygous deletion of *IHH* in the Short-legged population.

We established parental crosses with Short-legged individuals and selected lethal phenotypes based on morphological traits for whole-genome resequencing. Alignment using IGV revealed that lethal samples exhibited two deletions within the regions from 22,287,390–22,300,869 bp and 22,301,448–22,313,006 bp. These two non-contiguous deletions (25,039 bp in total) span intron 4 to exon 7 of the *NHEJ1* gene and the entire *IHH* gene, slightly exhibiting different size compared to Japanese bantam chicken [11] (25,044 bp in total). Notably, the presence of a 578 bp inversion within the intergenic region has not been previously described in any Chinese short-legged chicken breed, representing a unique structural feature of the Yunlong Short-Leg chicken mutation.

This inversion may potentially affect the regulatory landscape of the IHH-NHEJ1 locus, although this hypothesis requires further functional investigation. qRT-PCR was used to quantify relative mRNA expression of *NHEJ1* and *IHH* in different genotypes. The results revealed that expression levels of *IHH* and *NHEJ1* were significantly higher in Long-legged samples than in Short-legged samples and were undetectable in lethal samples, consistent with the observed gene deletions. Importantly, future studies using stage-matched samples (e.g., E4 limb buds from all genotypes) are needed to rigorously quantify the homozygous mutation effect. Non-homologous end joining 1 (NHEJ1, also known as XLF) is a key protein involved in the non-homologous end joining (NHEJ) pathway, which repairs DNA double-strand breaks (DSBs). It is termed “non-homologous” because it joins DNA ends without a homologous template, unlike homology-directed repair (HDR), which relies on sequence homology [42]. Studies using NHEJ1-deficient human induced pluripotent stem cells have demonstrated that the absence of *NHEJ1* impairs DSB repair capacity, making the cells vulnerable to physiological stress, metabolic imbalances, and radiation-induced DNA damage [43].

Kinoshita et al. [11] showed that *NHEJ1* is essential for DSB repair in chicken embryos. In Cp mutant embryos, early embryonic death is associated with defective DSB repair in the brain and neural tube. However, this differs from the Xingyi Short-legged chicken Cp mutants, which exhibit only *IHH* deletion without *NHEJ1* loss [10]. Thus, *IHH* deletion alone can induce the Creeper phenotype, but the precise mutation fragments in homozygous Cp alleles may vary across Short-legged chicken breeds.

We acknowledge that the sample size (*n* = 60) used in the GWAS is relatively small, which may reduce statistical power and increase the risk of false negatives. This is a limitation of the present study, primarily due to the limited population size of the rare Yunlong Short-Leg chicken breed. Although both males and females were included in the GWAS with sex as a covariate, we acknowledge that the sample size precludes sex-stratified analyses. Future studies with larger cohorts are needed to validate and refine these results, and to explore potential sex-specific genetic effects on shank length.

Our findings suggest that Short-legged individuals in the Yunlong Short-Leg chicken population exhibit the Cp phenotype, and the combined deletion of *IHH* and *NHEJ1* serves as the likely genetic basis. These results offer valuable insights into the genetic mechanisms underlying shortened shank length in Short-legged chicken breeds.

## Conclusion

Population structure analysis indicated that Long-legged and Short-legged Yunlong Short-Leg chickens share the same genetic background without obvious stratification, confirming that they belong to a single population. Both Short-legged and lethal Yunlong Short-Leg samples exhibited a 25,039 bp deletion and a 578 bp inversion on chromosome 7. The two non-contiguous deletions span intron 4 to exon 7 of the *NHEJ1* gene and the entire *IHH* gene, while the inversion is located in the intergenic region between *IHH* and *NHEJ1*. No such variations were observed in Long-legged samples. Multiplex PCR effectively distinguished genotypes associated with different shank phenotypes. Mutations in *IHH* and *NHEJ1* are strongly associated with the variation in shank length in Yunlong Short-Leg chickens.

## Supplementary Information

Supplementary Material 1.

## Data Availability

The raw sequencing data generated from 66 chickens in this study have been deposited in the National Genomics Data Center (NGDC) BioProject database under the accession number PRJCA026306. Data generated or analysed during this study are included in this published article and its supplementary information files. We declare that the data supporting the findings of this study are available within the article and its supplementary information files. #For review convenience, we have generated a link through which reviewers can access the raw data ( https://ngdc.cncb.ac.cn/gsa/s/woo38QV3 , valid for two months, before April 27, 2026).

## References

[1] Boegheim IJM, Leegwater PaJ, Van Lith HA, et al. Current insights into the molecular genetic basis of dwarfism in livestock[J]. Vet J. 2017;224:64–75. 28697878 10.1016/j.tvjl.2017.05.014

[2] Cutler IE. Reptilian fowls: a study in atavistic heredity. J Hered. 1925;16(10):353–6.

[3] Cole RK. An autosomal dwarfism in the domestic fowl. Poult Sci. 2000;79(11):1507–16. 11092317 10.1093/ps/79.11.1507

[4] Ferdaus AJ, Bhuiyan M, Bhuiyan A K F, H, et al. Body conformation, morphometry indices and inheritance pattern of indigenous dwarf chickens of Bangladesh. J Poult Res. 2019;16(2):55–61.

[5] Wu Z. Small chicken, big story: detection of the genetic background of dwarfism in chicken using genomic analyses[D]. Wageningen: Wageningen University; 2021.

[6] Perini F, Cendron F, Wu Z, et al. Genomics of dwarfism in italian local chicken breeds. Genes (Basel). 2023;14(3):633. 10.3390/genes14030633 PMC10047989 36980905

[7] Wu Z, Bortoluzzi C, Derks MFL, et al. Heterogeneity of a dwarf phenotype in Dutch traditional chicken breeds revealed by genomic analyses. Evol Appl. 2021;14(4):1095–108. 33897823 10.1111/eva.13183 PMC8061282

[8] Wu Z, Derks MFL, Dibbits B, et al. A novel loss-of-function variant in transmembrane protein 263 (TMEM263) of autosomal dwarfism in chicken. Front Genet. 2018;9:193. 29930570 10.3389/fgene.2018.00193 PMC6001002

[9] Landauer W. Studies on the creeper fowl. J Genet. 1932;25:367–94.

[10] Jin S, Zhu F, Wang Y, et al. Deletion of Indian hedgehog gene causes dominant semi-lethal Creeper trait in chicken. Sci Rep. 2016;6:30172. 27439785 10.1038/srep30172 PMC4954956

[11] Kinoshita K, Suzuki T, Koike M, et al. Combined deletions of IHH and NHEJ1 cause chondrodystrophy and embryonic lethality in the Creeper chicken. Commun Biol. 2020;3(1):144. 32214226 10.1038/s42003-020-0870-z PMC7096424

[12] Alsoufi MA, Changrong G. Genetic Diversity and Evolution of Yunnan Chicken Breeds of China. In: Maia RT, Campos MDA, editors. Population Genetics. London: IntechOpen; 2022. 10.5772/intechopen.102915.

[13] NY/T 823–2020; performance terminology and measurements for poultry. Beijing: China standards press; 2020.

[14] Chen S, Zhou Y, Chen Y, et al. fastp: an ultra-fast all-in-one FASTQ preprocessor. Bioinformatics. 2018;34(17):i884–90. 30423086 10.1093/bioinformatics/bty560 PMC6129281

[15] Li H, Durbin R. Fast and accurate long-read alignment with Burrows-Wheeler transform[J]. Bioinformatics. 2010;26(5):589–95. 20080505 10.1093/bioinformatics/btp698 PMC2828108

[16] Li H, Handsaker B, Wysoker A, et al. The sequence alignment/map format and SAMtools. Bioinformatics. 2009;25(16):2078–9. 19505943 10.1093/bioinformatics/btp352 PMC2723002

[17] Mckenna A, Hanna M, Banks E, et al. The genome analysis toolkit: a MapReduce framework for analyzing next-generation DNA sequencing data. Genome Res. 2010;20(9):1297–303. 20644199 10.1101/gr.107524.110 PMC2928508

[18] Wang K, Li M, Hakonarson H. ANNOVAR: functional annotation of genetic variants from high-throughput sequencing data. Nucleic Acids Res. 2010;38(16):e164. 20601685 10.1093/nar/gkq603 PMC2938201

[19] Purcell S, Neale B, Todd-Brown K, et al. PLINK: a tool set for whole-genome association and population-based linkage analyses. Am J Hum Genet. 2007;81(3):559–75. 17701901 10.1086/519795 PMC1950838

[20] Lee TH, Guo H, Wang X, et al. SNPhylo: a pipeline to construct a phylogenetic tree from huge SNP data. BMC Genomics. 2014;15:162. 24571581 10.1186/1471-2164-15-162 PMC3945939

[21] Xie J, Chen Y, Cai G, et al. Tree Visualization By One Table (tvBOT): a web application for visualizing, modifying and annotating phylogenetic trees. Nucleic Acids Res. 2023;51(W1):W587–92. 37144476 10.1093/nar/gkad359 PMC10320113

[22] Alexander DH, Novembre J, Lange K. Fast model-based estimation of ancestry in unrelated individuals. Genome Res. 2009;19(9):1655–64. 19648217 10.1101/gr.094052.109 PMC2752134

[23] Zhou X, Stephens M. Genome-wide efficient mixed-model analysis for association studies. Nat Genet. 2012;44(7):821–4. 22706312 10.1038/ng.2310 PMC3386377

[24] Hutt FB. Sex-linked dwarfism in the fowl. Heredity. 1949;50:209–21.

[25] Upp CW. Further data on the inheritance of dwarfism in fowls. Poult Sci. 1934;13(3):157–65.

[26] Buhr RJ, Abbott UK, Abplanalp H, et al. Effects of the sex-linked dwarf gene (dw) on the expression of the muscular dystrophy gene (am) in chicken. J Hered. 1991;82(6):465–70. 1795099 10.1093/oxfordjournals.jhered.a111129

[27] Uffelmann E, Huang QQ, Munung NS, et al. Genome-wide association studies. Nat Reviews Methods Primers. 2021;1(1):59.

[28] Wang S, Wang Y, Li Y, et al. Genome-wide association study and selective sweep analysis reveal the genetic architecture of body weights in a chicken F(2) resource population. Front Vet Sci. 2022;9:875454. 35958311 10.3389/fvets.2022.875454 PMC9361851

[29] Zhao X, Nie C, Zhang J, et al. Identification of candidate genomic regions for chicken egg number traits based on genome-wide association study. BMC Genomics. 2021;22(1):610. 34376144 10.1186/s12864-021-07755-3 PMC8356427

[30] Nowlan NC, Prendergast PJ, Murphy P. Identification of mechanosensitive genes during embryonic bone formation. PLoS Comput Biol. 2008;4(12):e1000250. 19112485 10.1371/journal.pcbi.1000250 PMC2592698

[31] Zhong C, Li X, Guan D, et al. Age-dependent genetic architectures of chicken body weight explored by multidimensional GWAS and molQTL analyses. J Genet Genomics. 2024;51(12):1423–34. 39306327 10.1016/j.jgg.2024.09.003

[32] Wang J, Liu J, Lei Q, et al. Elucidation of the genetic determination of body weight and size in Chinese local chicken breeds by large-scale genomic analyses. BMC Genomics. 2024;25(1):296. 38509464 10.1186/s12864-024-10185-6 PMC10956266

[33] Saikia A, Sarma HN. Role of Ihh — a progesterone-responsive gene in mammalian reproduction: a review. Middle East Fertility Soc J. 2024;29(1):48. 10.1186/s43043-024-00206-5.

[34] Wu M, Chen G, Li YP. TGF-beta and BMP signaling in osteoblast, skeletal development, and bone formation, homeostasis and disease. Bone Res. 2016;4:16009. 27563484 10.1038/boneres.2016.9 PMC4985055

[35] Vasques GA, Funari MFA, Ferreira FM, et al. IHH gene mutations causing short stature with nonspecific skeletal abnormalities and response to growth hormone therapy. J Clin Endocrinol Metab. 2018;103(2):604–14. 29155992 10.1210/jc.2017-02026

[36] Li L, Su H, Lei J, et al. Missense variant c.298G > T in the IHH gene: expanding the phenotypic spectrum of Brachydactyly type A1. Gene Rep. 2025;38:102116. 10.1016/j.genrep.2024.102116.

[37] Zhu T, Guan L, Chen D, et al. A novel heterozygous IHH c.331_333del mutation identified in a fetus with brachydactyly type A1 causes IHH protein maturation failure in HEK293T cells. Phenomics. 2024;5(2):123–36. 40606564 10.1007/s43657-024-00191-9 PMC12209099

[38] Chen Y, Yin M, Lu Y, et al. Short stature with brachydactyly caused by a novel mutation in the IHH gene and response to 4-year growth hormone therapy: a case report. Transl Pediatr. 2024;13(5):856–63. 38840672 10.21037/tp-23-578 PMC11148735

[39] Zhang R, Cong F, Li Q, Min Z, Yan J, Zhang Q, Ma J, Lu S, Ma J. miR-497 is implicated in the process of chondrogenesis and inhibits IHH gene expression in human chondrocytes. Cartilage. 2018;11:479–89. 30156864 10.1177/1947603518796126 PMC7488943

[40] Imsland F, Feng C, Boije H, et al. The Rose-comb mutation in chickens constitutes a structural rearrangement causing both altered comb morphology and defective sperm motility. PLoS Genet. 2012;8(6):e1002775. 22761584 10.1371/journal.pgen.1002775 PMC3386170

[41] Landauer W. Creeper and single-comb linkage in fowl. Nature. 1933;132(3337):606–606.

[42] Andres SN, Vergnes A, Ristic D, et al. A human XRCC4-XLF complex bridges DNA. Nucleic Acids Res. 2012;40(4):1868–78. 22287571 10.1093/nar/gks022 PMC3287209

[43] Tilgner K, Neganova I, Singhapol C, et al. Brief report: a human induced pluripotent stem cell model of cernunnos deficiency reveals an important role for XLF in the survival of the primitive hematopoietic progenitors. Stem Cells. 2013;31(9):2015–23. 23818183 10.1002/stem.1456

