# Supplementary material for: A heterozygous deletion and inversion at the NHEJ1‑IHH locus associated with shank length in Yunlong short-leg chicken
Source: BMC Genomics. 2026 May 18;27:607. doi: 10.1186/s12864-026-12943-0 (PMC13352712; doi:10.1186/s12864-026-12943-0)

**Supplementary Figures**

**Supplementary Figure 1.** Haplotype analysis of SNPs in GWAS candidate regions.


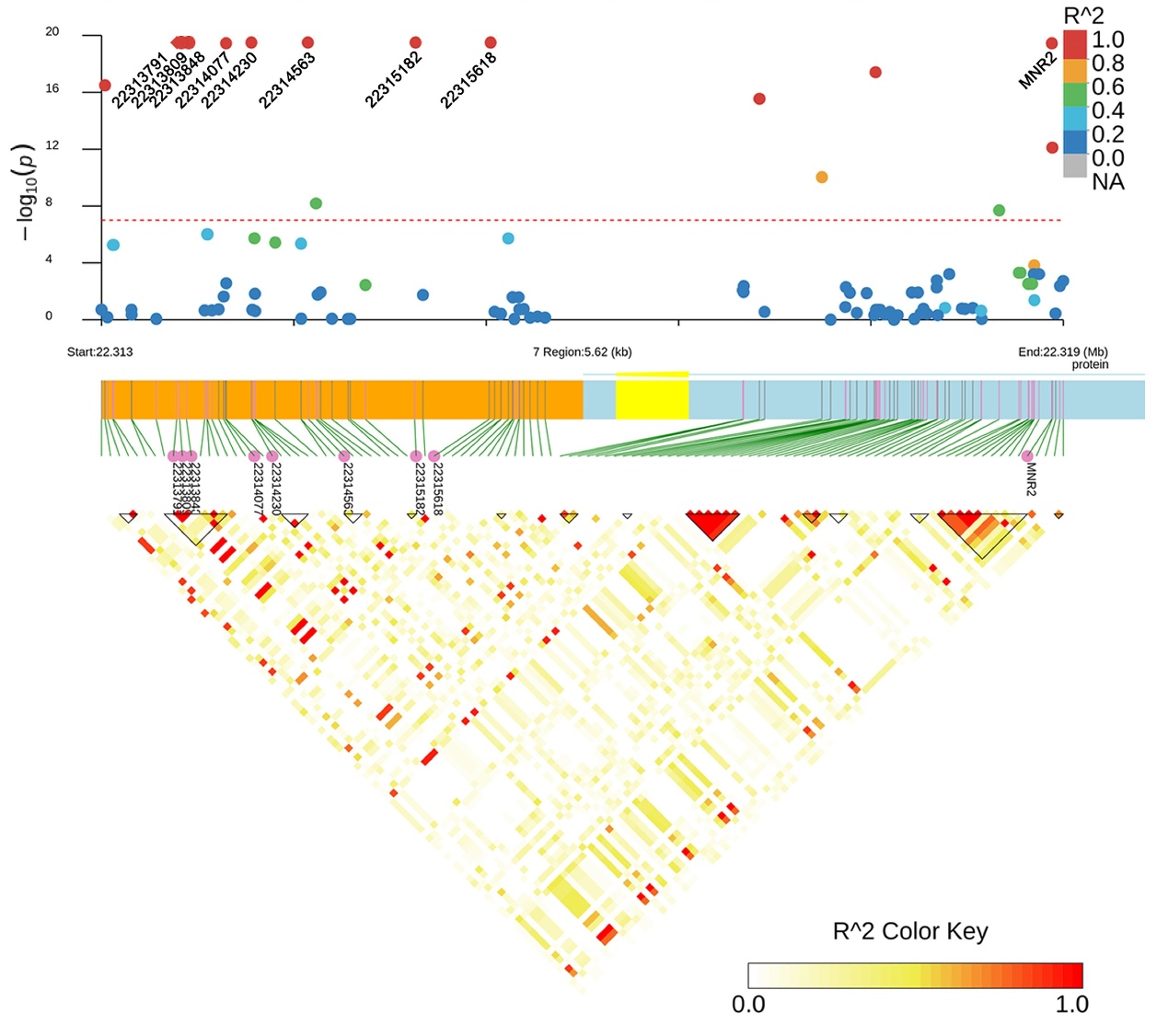


**Supplementary Figure 2.** Dissection characteristics of Yunlong Short-Leg chicken embryo eggs at E4 stage.


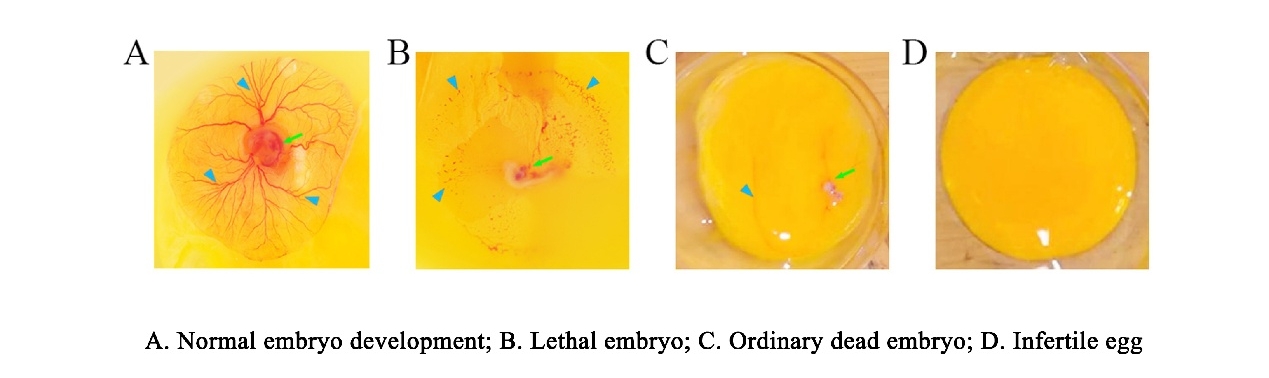


**Supplementary Figure 3.** Sequencing results of lethal samples in GWAS candidate regions.


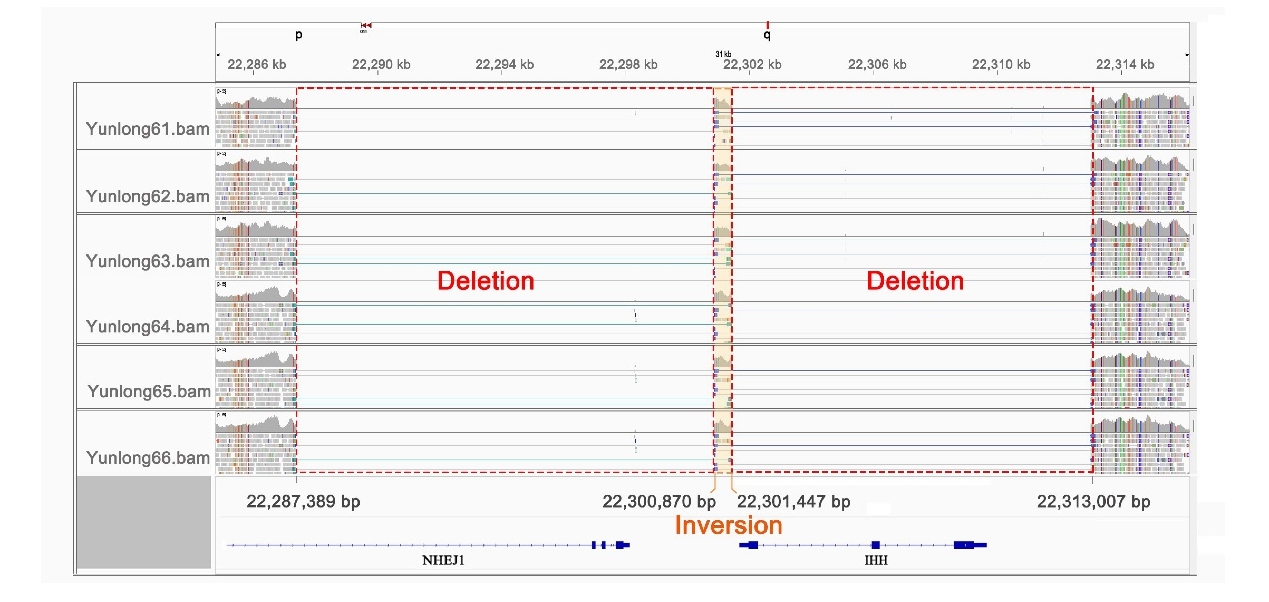


**Supplementary Figure 4.** Amplification of mutation regions in the genome of Yunlong Short-Leg chickens of different genotypes.


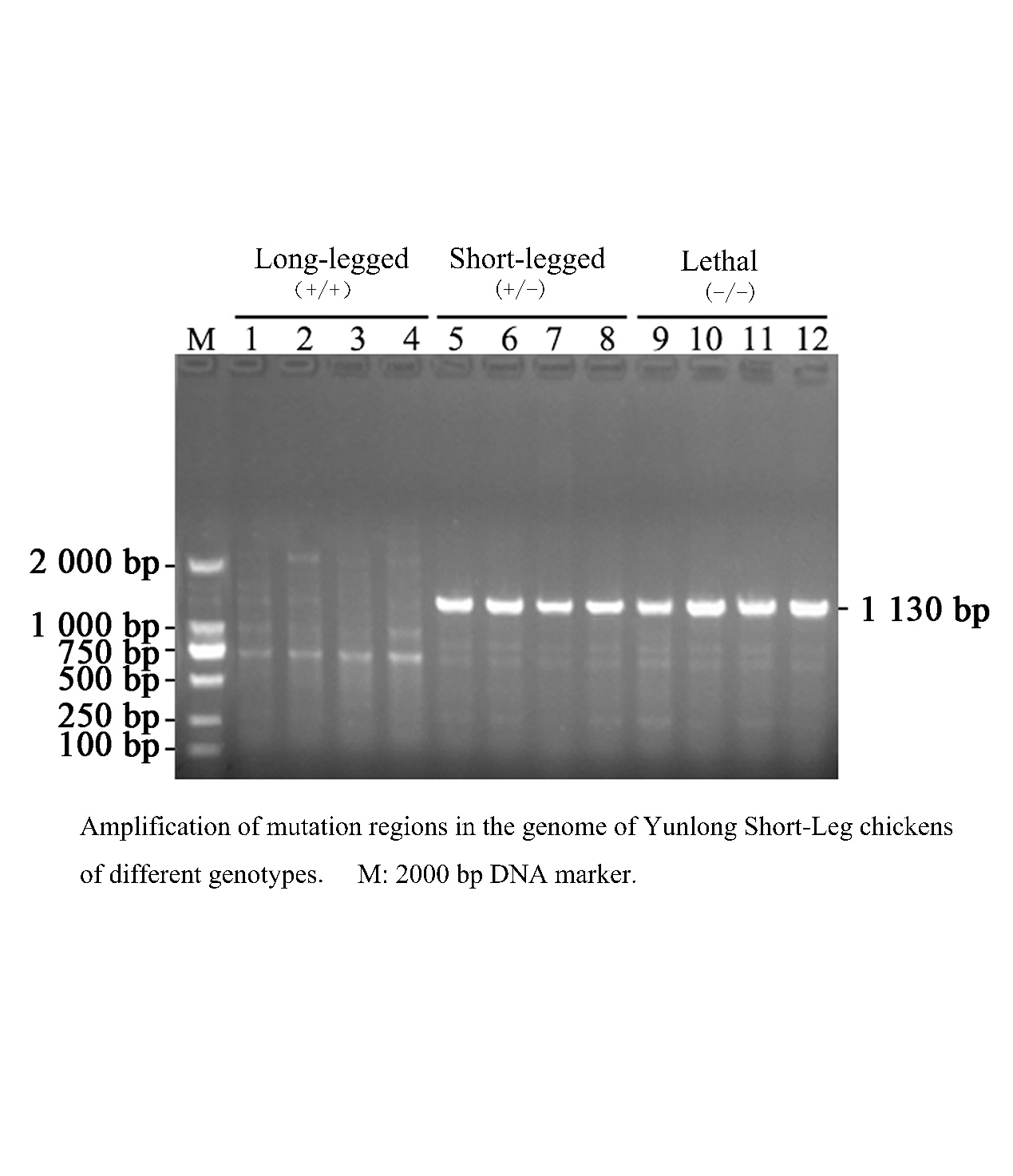


**Supplementary Figure 5.** Sequencing results of PCR amplification products of Yunlong Short-Leg chicken.


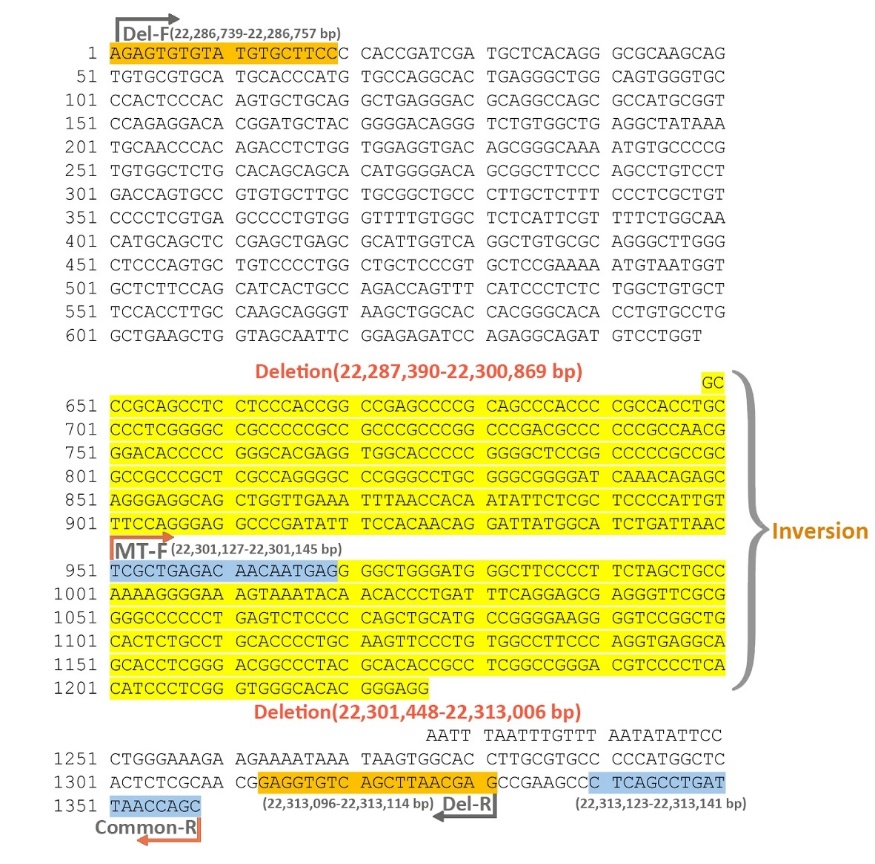


**Supplementary Figure 6.** Original PCR gel image of Figure 5 and Supplementary Figure 4.


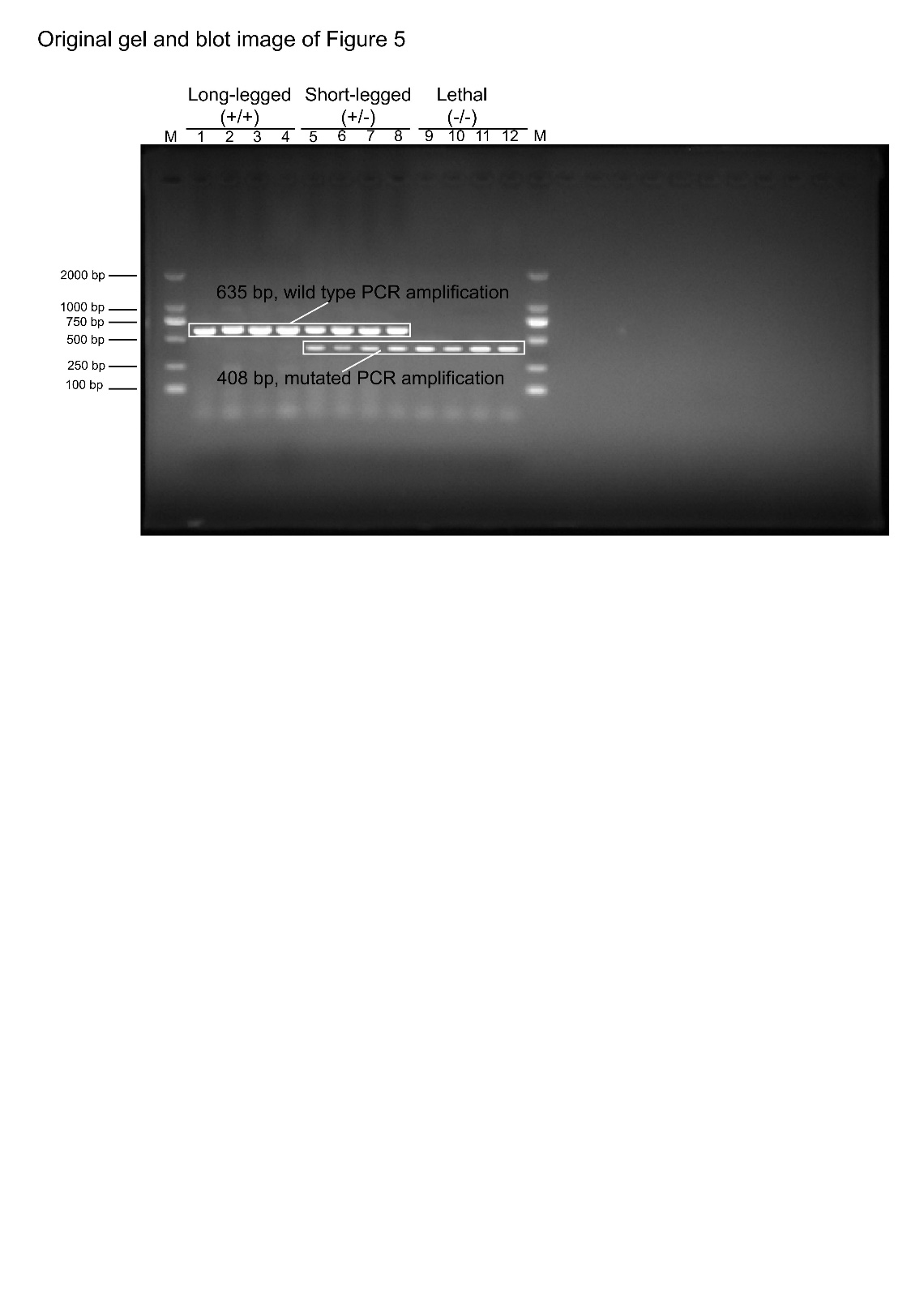


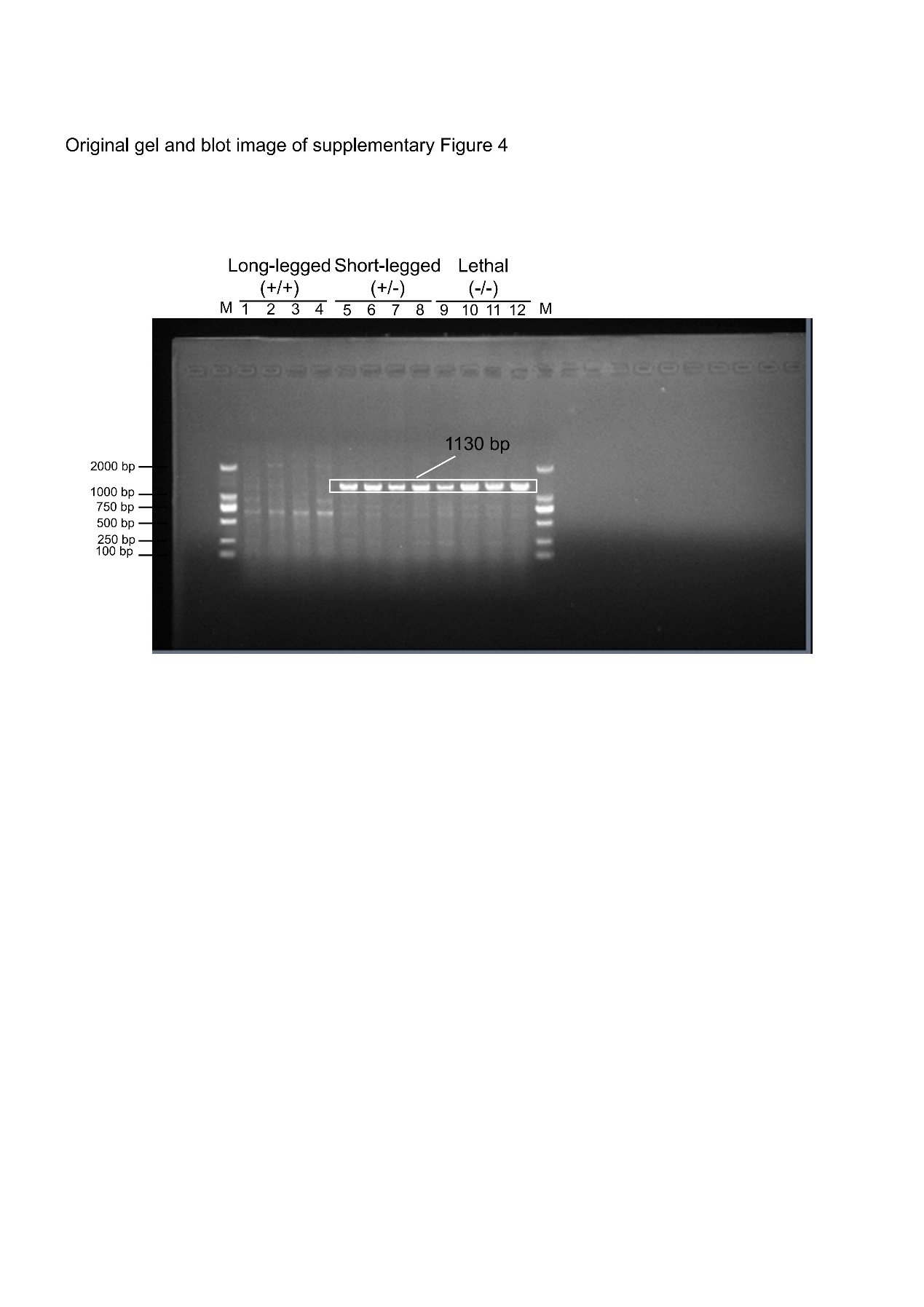

Supplement: Supplementary file 1 — Supplementary Material 1. [file 12864_2026_12943_MOESM1_ESM.zip › Supplementary Figures.docx]
