# Supplementary material for: A heterozygous deletion and inversion at the NHEJ1‑IHH locus associated with shank length in Yunlong short-leg chicken
Source: BMC Genomics. 2026 May 18;27:607. doi: 10.1186/s12864-026-12943-0 (PMC13352712; doi:10.1186/s12864-026-12943-0)
Supplement: Supplementary file 1 — Supplementary Material 1. [file 12864_2026_12943_MOESM1_ESM.zip › Supplementary Information Tables.docx]

| **Supplementary Table 1. RT-qPCR amplification primer sequences** | | |
| --- | --- | --- |
| **Genes** | **Primer sequences (5’→3’)** | **Product length (bp)** |
| *IHH* | F：GCCATCTCCGTCATGAACCA | 146 |
|  | R：TACTTGTTGCGGTCCCTGTC |  |
| *NHEJ*1 | F：AGAACATCATGGCTGAGAGCCT | 262 |
|  | R：TTCTGCACTGGGGAGGAGGT |  |
| *MNR*2 | F：CACTGAGACGCAGGTGAAGA | 141 |
|  | R：CTGGGCAGCAGACTCTCATC |  |
| *DNPEP* | F：GAACAGCAATCAGCGCTACG | 101 |
|  | R：TGCGTACCATGAACTCCTGC |  |
| *FAM*134A | F：CATGGCCACGACCGCC | 229 |
|  | R：GGAGGAAAACAACCAGAGCG |  |
| SLC23A3 | F：CCTGAACCGCACTGTACCAT | 193 |
|  | R：CCTCCTTGTAGGCAGACAGC |  |
| *CNPPD*1 | F：GTGTCTCATGTGTCTCGGGAA | 246 |
|  | R：CTCCAGCGTGTTCATGGTCT |  |
| *FEV* | F：CTGCCCTAACCTGAAGGGAT | 263 |
|  | R：CAGCTCCGATGTTTTTGCGT |  |
| *β-actin* | F：GAGAAATTGTGCGTGACATCA | 152 |
|  | R：CCTGAACCTCTCATTGCCA |  |

| **Supplementary Table 2. Yunlong Short Leg chicken weight and body size characteristics** | | | | | | | | | | | |
| --- | --- | --- | --- | --- | --- | --- | --- | --- | --- | --- | --- |
| **Sample** | **Phenotype** | **Sex** | **Body weight（kg）** | **Body slant length（cm)** | **Fossil bone length（cm）** | **Shank Length（mm)** | **Shank Circumference（cm）** | **Breast depth（mm)** | **Breast width（mm)** | **Pelvis width（mm）** | **Breast angle（°）** |
| Yunlong1 | Short-legged Rooster | Male | 1.593 | 18.00 | 10.00 | 68.22 | 3.2 | 108.70 | 89.11 | 64.88 | 114.00 |
| Yunlong2 | Short-legged Rooster | Male | 2.065 | 18.00 | 11.00 | 66.46 | 4.3 | 108.76 | 101.54 | 80.89 | 92.16 |
| Yunlong3 | Short-legged Rooster | Male | 1.762 | 20.00 | 11.00 | 68.17 | 4.0 | 107.84 | 101.77 | 68.35 | 86.30 |
| Yunlong4 | Short-legged Rooster | Male | 2.405 | 22.60 | 10.60 | 79.16 | 4.7 | 109.96 | 112.22 | 68.21 | 94.00 |
| Yunlong5 | Short-legged Rooster | Male | 2.307 | 19.60 | 10.00 | 73.40 | 5.4 | 77.87 | 99.85 | 65.32 | 103.00 |
| Yunlong6 | Short-legged Rooster | Male | 1.833 | 22.20 | 9.80 | 66.13 | 4.0 | 99.66 | 104.08 | 65.55 | 98.00 |
| Yunlong7 | Short-legged Rooster | Male | 2.381 | 21.50 | 10.30 | 68.78 | 5.2 | 102.09 | 86.33 | 71.76 | 103.50 |
| Yunlong8 | Short-legged Rooster | Male | 1.891 | 21.60 | 10.10 | 61.97 | 4.0 | 113.27 | 96.68 | 73.48 | 99.10 |
| Yunlong9 | Short-legged Rooster | Male | 2.156 | 21.70 | 10.90 | 73.16 | 4.1 | 116.02 | 88.00 | 74.97 | 96.00 |
| Yunlong10 | Short-legged Rooster | Male | 1.900 | 21.60 | 10.90 | 65.69 | 4.2 | 119.78 | 94.33 | 70.03 | 100.20 |
| Yunlong11 | Short-legged Rooster | Male | 1.840 | 21.20 | 10.30 | 66.49 | 4.8 | 117.59 | 89.88 | 64.54 | 102.40 |
| Yunlong12 | Short-legged Rooster | Male | 2.604 | 21.80 | 11.10 | 74.33 | 4.7 | 128.93 | 87.74 | 78.46 | 100.00 |
| Yunlong13 | Short-legged Rooster | Male | 2.516 | 21.80 | 11.40 | 79.89 | 5.2 | 136.15 | 105.40 | 75.07 | 96.40 |
| Yunlong14 | Short-legged Rooster | Male | 2.198 | 20.90 | 11.60 | 69.56 | 4.8 | 125.86 | 96.50 | 64.69 | 101.38 |
| Yunlong15 | Short-legged Rooster | Male | 2.423 | 27.00 | 11.30 | 75.65 | 5.0 | 97.06 | 96.34 | 75.98 | 114.00 |
| Yunlong16 | Short-legged Rooster | Male | 2.011 | 25.40 | 11.80 | 73.34 | 5.0 | 101.96 | 80.03 | 62.16 | 95.30 |
| Yunlong17 | Short-legged Hen | Female | 2.162 | 20.30 | 9.30 | 60.19 | 4.3 | 98.53 | 84.85 | 59.22 | 122.46 |
| Yunlong18 | Short-legged Hen | Female | 1.572 | 20.70 | 9.10 | 59.37 | 3.8 | 97.93 | 80.44 | 63.16 | 119.00 |
| Yunlong19 | Short-legged Hen | Female | 1.601 | 17.90 | 10.40 | 57.84 | 3.6 | 97.59 | 67.81 | 54.75 | 101.20 |
| Yunlong20 | Short-legged Hen | Female | 2.002 | 19.90 | 9.60 | 59.04 | 3.7 | 109.45 | 64.04 | 56.27 | 97.46 |
| Yunlong21 | Short-legged Hen | Female | 1.949 | 21.10 | 10.20 | 61.59 | 3.7 | 94.54 | 78.55 | 56.53 | 92.00 |
| Yunlong22 | Short-legged Hen | Female | 2.097 | 20.30 | 10.50 | 64.20 | 4.0 | 115.19 | 68.23 | 56.04 | 95.00 |
| Yunlong23 | Short-legged Hen | Female | 1.510 | 20.30 | 10.00 | 57.58 | 3.2 | 89.47 | 64.49 | 53.53 | 88.00 |
| Yunlong24 | Short-legged Hen | Female | 1.379 | 20.30 | 9.00 | 54.55 | 3.6 | 90.48 | 67.12 | 51.11 | 98.00 |
| Yunlong25 | Short-legged Hen | Female | 1.194 | 20.50 | 9.00 | 51.74 | 3.4 | 88.55 | 60.97 | 52.54 | 102.00 |
| Yunlong26 | Short-legged Hen | Female | 1.620 | 20.30 | 9.80 | 60.18 | 5.7 | 101.57 | 62.57 | 50.00 | 88.34 |
| Yunlong27 | Short-legged Hen | Female | 1.910 | 20.50 | 9.90 | 64.73 | 4.1 | 97.32 | 78.10 | 51.77 | 107.30 |
| Yunlong28 | Short-legged Hen | Female | 1.898 | 19.50 | 8.40 | 64.63 | 3.2 | 95.41 | 68.75 | 64.91 | 100.00 |
| Yunlong29 | Short-legged Hen | Female | 1.766 | 20.80 | 8.80 | 69.29 | 4.0 | 98.97 | 66.27 | 57.73 | 97.40 |
| Yunlong30 | Short-legged Hen | Female | 1.526 | 20.30 | 9.00 | 60.57 | 3.7 | 82.38 | 81.87 | 53.22 | 97.00 |
| Yunlong31 | Short-legged Hen | Female | 1.775 | 17.60 | 9.10 | 65.08 | 4.1 | 72.27 | 90.84 | 55.26 | 100.40 |
| Yunlong32 | Short-legged Hen | Female | 1.470 | 22.90 | 9.50 | 55.79 | 3.3 | 98.97 | 69.34 | 58.73 | 95.10 |
| Yunlong33 | Long-legged Rooster | Male | 2.765 | 26.70 | 12.00 | 98.70 | 5.0 | 106.42 | 85.77 | 52.81 | 118.00 |
| Yunlong34 | Long-legged Rooster | Male | 2.152 | 24.50 | 11.50 | 97.35 | 4.3 | 119.29 | 94.83 | 58.20 | 128.10 |
| Yunlong35 | Long-legged Rooster | Male | 1.371 | 27.70 | 10.70 | 109.26 | 5.2 | 127.26 | 100.32 | 48.80 | 120.00 |
| Yunlong36 | Long-legged Rooster | Male | 2.267 | 23.60 | 9.40 | 95.69 | 4.6 | 120.71 | 84.36 | 54.94 | 113.40 |
| Yunlong37 | Long-legged Rooster | Male | 2.685 | 29.60 | 10.70 | 87.80 | 5.4 | 108.28 | 98.55 | 61.83 | 122.00 |
| Yunlong38 | Long-legged Rooster | Male | 2.794 | 29.40 | 12.70 | 102.59 | 4.7 | 94.05 | 85.65 | 74.38 | 120.00 |
| Yunlong39 | Long-legged Rooster | Male | 2.302 | 27.70 | 10.60 | 93.35 | 4.5 | 130.25 | 87.69 | 62.09 | 115.00 |
| Yunlong40 | Long-legged Rooster | Male | 2.527 | 27.40 | 11.50 | 104.98 | 4.8 | 115.79 | 111.49 | 60.22 | 112.00 |
| Yunlong41 | Long-legged Rooster | Male | 2.327 | 26.80 | 11.20 | 91.04 | 3.8 | 111.94 | 82.86 | 59.62 | 99.50 |
| Yunlong42 | Long-legged Rooster | Male | 2.047 | 26.40 | 11.20 | 91.86 | 4.3 | 110.15 | 89.18 | 59.99 | 105.50 |
| Yunlong43 | Long-legged Rooster | Male | 2.944 | 28.70 | 12.20 | 94.58 | 5.4 | 120.98 | 104.05 | 67.96 | 114.40 |
| Yunlong44 | Long-legged Rooster | Male | 2.437 | 27.30 | 12.20 | 90.81 | 4.5 | 116.72 | 94.07 | 61.16 | 115.20 |
| Yunlong45 | Long-legged Rooster | Male | 2.147 | 26.00 | 10.50 | 80.34 | 4.3 | 100.47 | 86.67 | 58.13 | 125.10 |
| Yunlong46 | Long-legged Rooster | Male | 2.491 | 27.70 | 11.30 | 99.05 | 5.5 | 132.58 | 88.97 | 65.24 | 102.20 |
| Yunlong47 | Long-legged Hen | Female | 1.541 | 22.00 | 9.70 | 79.14 | 3.7 | 111.22 | 74.96 | 49.12 | 84.50 |
| Yunlong48 | Long-legged Hen | Female | 2.541 | 24.60 | 11.70 | 76.43 | 4.4 | 104.51 | 93.49 | 55.41 | 84.10 |
| Yunlong49 | Long-legged Hen | Female | 1.611 | 21.70 | 8.60 | 73.34 | 3.6 | 94.02 | 71.26 | 61.30 | 88.50 |
| Yunlong50 | Long-legged Hen | Female | 1.727 | 22.80 | 9.20 | 73.01 | 3.4 | 104.87 | 82.58 | 55.54 | 106.10 |
| Yunlong51 | Long-legged Hen | Female | 1.796 | 26.30 | 9.60 | 80.90 | 4.2 | 108.60 | 78.68 | 55.95 | 105.40 |
| Yunlong52 | Long-legged Hen | Female | 1.925 | 21.70 | 10.80 | 87.99 | 4.0 | 112.51 | 74.90 | 53.72 | 88.00 |
| Yunlong53 | Long-legged Hen | Female | 2.128 | 22.50 | 10.30 | 83.48 | 4.5 | 113.13 | 81.27 | 62.34 | 86.00 |
| Yunlong54 | Long-legged Hen | Female | 1.732 | 21.50 | 8.70 | 78.00 | 3.4 | 103.19 | 72.20 | 52.07 | 99.40 |
| Yunlong55 | Long-legged Hen | Female | 1.656 | 23.60 | 9.20 | 74.20 | 3.4 | 103.72 | 73.71 | 55.13 | 101.10 |
| Yunlong56 | Long-legged Hen | Female | 1.933 | 24.40 | 9.50 | 85.06 | 4.0 | 93.35 | 78.60 | 56.31 | 97.00 |
| Yunlong57 | Long-legged Hen | Female | 2.028 | 22.20 | 9.80 | 87.67 | 3.9 | 120.31 | 76.00 | 54.26 | 97.00 |
| Yunlong58 | Long-legged Hen | Female | 2.153 | 21.10 | 9.40 | 80.27 | 3.6 | 104.33 | 82.93 | 57.38 | 118.50 |
| Yunlong59 | Long-legged Hen | Female | 1.439 | 23.00 | 10.00 | 81.21 | 2.7 | 92.73 | 66.57 | 48.97 | 85.00 |
| Yunlong60 | Long-legged Hen | Female | 2.262 | 26.00 | 10.60 | 84.63 | 4.4 | 109.70 | 86.48 | 60.10 | 91.00 |

| **Supplementary Table 3. Whole genome quality control information table** | | | | | | | |
| --- | --- | --- | --- | --- | --- | --- | --- |
| Sample_ID | Raw reads | Clean reads | Raw bases(G) | Clean_bases(G) | Clean_Q20(%) | Clean_Q30(%) | Clean_GC_rate(%) |
| Yunlong1 | 150746216 | 150746214 | 19.37 | 19.35 | 91.16% | 74.94% | 43.93% |
| Yunlong2 | 143771106 | 143771106 | 19.88 | 19.87 | 97.39% | 92.15% | 44.12% |
| Yunlong3 | 152292268 | 152292268 | 20.94 | 20.93 | 97.93% | 93.71% | 43.25% |
| Yunlong4 | 162941100 | 162941100 | 22.56 | 22.55 | 96.79% | 90.55% | 44.19% |
| Yunlong5 | 142178000 | 142178000 | 19.84 | 19.83 | 97.35% | 92.09% | 44.07% |
| Yunlong6 | 131232500 | 131232500 | 18.35 | 18.34 | 96.76% | 90.37% | 44.42% |
| Yunlong7 | 147377422 | 147377422 | 20.38 | 20.37 | 96.89% | 90.73% | 43.98% |
| Yunlong8 | 154800030 | 154800030 | 21.42 | 21.41 | 97.55% | 92.67% | 43.81% |
| Yunlong9 | 146169386 | 146169386 | 20.44 | 20.44 | 96.15% | 88.52% | 44.65% |
| Yunlong10 | 218258000 | 218258000 | 30.65 | 30.64 | 97.60% | 92.73% | 44.20% |
| Yunlong11 | 230809122 | 230809122 | 32.63 | 32.62 | 97.61% | 92.78% | 43.92% |
| Yunlong12 | 186574808 | 186574808 | 26.54 | 26.53 | 97.76% | 93.23% | 44.83% |
| Yunlong13 | 153497968 | 153497968 | 21.93 | 21.92 | 96.32% | 89.04% | 44.74% |
| Yunlong14 | 325687012 | 325687012 | 45.50 | 45.48 | 97.38% | 92.08% | 44.18% |
| Yunlong15 | 237085130 | 237085128 | 33.16 | 33.15 | 97.58% | 92.72% | 43.90% |
| Yunlong16 | 173323822 | 173323822 | 24.45 | 24.44 | 97.69% | 93.06% | 44.29% |
| Yunlong17 | 158160208 | 158160206 | 21.95 | 21.94 | 96.18% | 88.72% | 44.10% |
| Yunlong18 | 192424980 | 192424980 | 26.68 | 26.66 | 97.29% | 91.91% | 44.12% |
| Yunlong19 | 144908602 | 144908602 | 20.18 | 20.17 | 97.22% | 91.68% | 44.16% |
| Yunlong20 | 154279688 | 154279688 | 21.33 | 21.32 | 96.83% | 90.59% | 44.12% |
| Yunlong21 | 156528234 | 156528234 | 21.88 | 21.88 | 97.28% | 91.87% | 44.08% |
| Yunlong22 | 146622278 | 146622278 | 20.34 | 20.33 | 96.82% | 90.52% | 44.07% |
| Yunlong23 | 131776620 | 131776620 | 18.03 | 18.02 | 94.92% | 85.32% | 44.18% |
| Yunlong24 | 161420624 | 161420624 | 22.12 | 22.10 | 96.23% | 89.12% | 44.14% |
| Yunlong25 | 201192196 | 201192194 | 27.90 | 27.89 | 96.46% | 89.61% | 44.31% |
| Yunlong26 | 216450502 | 216450502 | 30.07 | 30.06 | 96.98% | 90.98% | 44.12% |
| Yunlong27 | 196769416 | 196769416 | 27.55 | 27.54 | 97.78% | 93.27% | 44.37% |
| Yunlong28 | 176190980 | 176190980 | 24.63 | 24.62 | 96.20% | 88.74% | 44.42% |
| Yunlong29 | 221508154 | 221508152 | 30.71 | 30.70 | 96.84% | 90.58% | 44.24% |
| Yunlong30 | 166389766 | 166389766 | 23.31 | 23.30 | 97.01% | 91.09% | 44.00% |
| Yunlong31 | 67923494 | 67923494 | 8.88 | 8.87 | 96.21% | 88.83% | 45.90% |
| Yunlong32 | 178422360 | 178422358 | 24.62 | 24.61 | 95.87% | 87.80% | 44.15% |
| Yunlong33 | 195256390 | 195256390 | 27.15 | 27.12 | 96.27% | 89.07% | 44.04% |
| Yunlong34 | 170281878 | 170281878 | 24.10 | 24.08 | 96.01% | 88.33% | 44.30% |
| Yunlong35 | 150234030 | 150234030 | 20.99 | 20.98 | 96.46% | 89.51% | 43.88% |
| Yunlong36 | 156162886 | 156162886 | 22.03 | 22.02 | 96.79% | 90.45% | 44.05% |
| Yunlong37 | 165055076 | 165055076 | 22.84 | 22.82 | 96.53% | 89.70% | 43.91% |
| Yunlong38 | 147597546 | 147597546 | 20.73 | 20.71 | 96.51% | 89.67% | 43.76% |
| Yunlong39 | 148089474 | 148089474 | 20.52 | 20.50 | 96.02% | 88.32% | 43.68% |
| Yunlong40 | 144198808 | 144198808 | 20.26 | 20.24 | 97.21% | 91.65% | 43.79% |
| Yunlong41 | 171751198 | 171751198 | 23.86 | 23.85 | 96.18% | 88.90% | 44.26% |
| Yunlong42 | 215460070 | 215460070 | 29.80 | 29.78 | 95.43% | 86.93% | 44.31% |
| Yunlong43 | 211987078 | 211987078 | 29.57 | 29.56 | 96.56% | 89.87% | 44.25% |
| Yunlong44 | 216112406 | 216112406 | 30.54 | 30.53 | 96.89% | 90.80% | 45.53% |
| Yunlong45 | 220927824 | 220927824 | 30.41 | 30.39 | 95.41% | 86.88% | 44.40% |
| Yunlong46 | 252803838 | 252803832 | 35.21 | 35.20 | 96.94% | 90.92% | 44.08% |
| Yunlong47 | 140682426 | 140682426 | 19.11 | 19.10 | 95.65% | 87.46% | 43.98% |
| Yunlong48 | 200603952 | 200603952 | 27.71 | 27.69 | 96.11% | 88.69% | 44.56% |
| Yunlong49 | 144228532 | 144228532 | 20.24 | 20.23 | 96.91% | 90.85% | 44.12% |
| Yunlong50 | 185935386 | 185935384 | 25.85 | 25.84 | 96.88% | 90.69% | 44.09% |
| Yunlong51 | 193867590 | 193867590 | 27.30 | 27.30 | 97.43% | 92.28% | 44.57% |
| Yunlong52 | 162279482 | 162279482 | 22.74 | 22.73 | 97.11% | 91.37% | 44.17% |
| Yunlong53 | 187007028 | 187007028 | 25.63 | 25.62 | 94.61% | 84.30% | 44.11% |
| Yunlong54 | 169533958 | 169533952 | 23.42 | 23.41 | 94.97% | 85.42% | 43.93% |
| Yunlong55 | 180104424 | 180104424 | 23.96 | 23.93 | 94.72% | 84.59% | 44.06% |
| Yunlong56 | 131887100 | 131887098 | 18.63 | 18.62 | 97.01% | 91.10% | 44.32% |
| Yunlong57 | 177896210 | 177896210 | 24.98 | 24.97 | 97.44% | 92.34% | 44.10% |
| Yunlong58 | 174180770 | 174180770 | 23.92 | 23.90 | 96.87% | 90.67% | 43.89% |
| Yunlong59 | 162600202 | 162600202 | 23.41 | 23.41 | 97.23% | 91.66% | 44.54% |
| Yunlong60 | 220305508 | 220305508 | 31.05 | 31.03 | 97.26% | 91.80% | 44.14% |
| Yunlong61 | 180413836 | 180404032 | 27.06 | 26.94 | 98.99% | 95.44% | 42.02% |
| Yunlong62 | 176403924 | 176394152 | 26.46 | 26.32 | 99.18% | 96.28% | 42.05% |
| Yunlong63 | 212621702 | 212590926 | 31.89 | 31.70 | 99.09% | 95.89% | 42.10% |
| Yunlong64 | 191354594 | 191344360 | 28.70 | 28.56 | 98.99% | 95.50% | 42.16% |
| Yunlong65 | 187052896 | 187047272 | 28.06 | 27.85 | 99.14% | 96.14% | 42.15% |
| Yunlong66 | 189845524 | 189833466 | 28.48 | 28.29 | 99.14% | 96.11% | 41.88% |

| **Supplementary Table 4. SNPs and InDels annotation statistical results** | | | |
| --- | --- | --- | --- |
| **SNPs annotation statistical results** | | | |
| Type |  | Number | Proportion(%） |
| downstream |  | 515144 | 2.44% |
| exonic | synonymous | 217963 | 1.67% |
|  | nonsynonymous | 130159 |  |
|  | unknown | 3372 |  |
|  | stopgain | 1466 |  |
|  | stoploss | 224 |  |
| exonic;splicing |  | 98 | 0.0005% |
| intergenic |  | 6920425 | 32.77% |
| intronic |  | 1E+07 | 49.07% |
| ncRNA_exonic |  | 419922 | 1.99% |
| ncRNA_exonic;splicing |  | 88 | 0.0004% |
| ncRNA_intronic |  | 1268180 | 6.01% |
| ncRNA_splicing |  | 626 | 0.0030% |
| splicing |  | 1072 | 0.01% |
| upstream |  | 507697 | 2.40% |
| upstream;downstream |  | 103131 | 0.49% |
| UTR3 |  | 435065 | 2.06% |
| UTR5 |  | 218843 | 1.04% |
| UTR5;UTR3 |  | 10132 | 0.05% |
| All |  | 2.1E+07 |  |
|  |  |  |  |
| **InDels annotation statistical results** | | | |
| **Type** | | **Number** | **Proportion(%**） |
| downstream | | 71082 | 2.42% |
| exonic | synonymous | 7424 | 0.42% |
|  | nonsynonymous | 4206 |  |
|  | unknown | 508 |  |
|  | stopgain | 130 |  |
|  | stoploss | 15 |  |
| exonic;splicing | | 29 | 0.001% |
| intergenic | | 984227 | 33.57% |
| intronic | | 1450278 | 49.47% |
| ncRNA_exonic | | 52211 | 1.78% |
| ncRNA_exonic;splicing | | 13 | 0.0004% |
| ncRNA_intronic | | 178834 | 6.10% |
| ncRNA_splicing | | 113 | 0.0039% |
| ncRNA_UTR5 | | 5 | 0.0002% |
| splicing | | 803 | 0.03% |
| upstream | | 66502 | 2.27% |
| upstream;downstream | | 15322 | 0.52% |
| UTR3 | | 70776 | 2.41% |
| UTR5 | | 27658 | 0.94% |
| UTR5;UTR3 | | 1560 | 0.05% |
| All | | 2931696 |  |

| **Supplementary Table 5. GWAS significant SNPs and InDels site annotation results** | | | | | |
| --- | --- | --- | --- | --- | --- |
| **GWAS significant SNPs site annotation results** | | | | | |
| rs | chr | ps | p_wald | type | annovar gene |
| 7:22177771 | 7 | 2.2E+07 | 1.84792E-15 | upstream | DNPEP (dist=409) |
| 7:22250315 | 7 | 2.2E+07 | 6.52418E-09 | intronic | FAM134A |
| 7:22251001 | 7 | 2.2E+07 | 7.37951E-11 | intronic | FAM134A |
| 7:22251002 | 7 | 2.2E+07 | 7.37951E-11 | intronic | FAM134A |
| 7:22251340 | 7 | 2.2E+07 | 4.35757E-13 | intronic | FAM134A |
| 7:22251646 | 7 | 2.2E+07 | 4.35757E-13 | intronic | FAM134A |
| 7:22252551 | 7 | 2.2E+07 | 1.00564E-09 | intronic | FAM134A |
| 7:22252683 | 7 | 2.2E+07 | 1.89459E-10 | intronic | FAM134A |
| 7:22252770 | 7 | 2.2E+07 | 1.02924E-10 | intronic | FAM134A |
| 7:22252789 | 7 | 2.2E+07 | 1.36497E-08 | intronic | FAM134A |
| 7:22252896 | 7 | 2.2E+07 | 1.36497E-08 | intronic | FAM134A |
| 7:22254529 | 7 | 2.2E+07 | 2.51782E-09 | intronic | CNPPD1 |
| 7:22254556 | 7 | 2.2E+07 | 7.02134E-14 | intronic | CNPPD1 |
| 7:22254742 | 7 | 2.2E+07 | 2.32464E-15 | exonic | CNPPD1 |
| 7:22254882 | 7 | 2.2E+07 | 2.15953E-13 | intronic | CNPPD1 |
| 7:22258857 | 7 | 2.2E+07 | 7.94594E-12 | exonic | CNPPD1 |
| 7:22260284 | 7 | 2.2E+07 | 4.79449E-09 | UTR5 | SLC23A3(rna-XM_015290080.2:c.-839T>C) |
| 7:22260303 | 7 | 2.2E+07 | 7.94594E-12 | UTR5 | SLC23A3(rna-XM_015290080.2:c.-820C>T) |
| 7:22260583 | 7 | 2.2E+07 | 7.94594E-12 | UTR5 | SLC23A3(rna-XM_015290080.2:c.-540C>T) |
| 7:22260629 | 7 | 2.2E+07 | 7.94594E-12 | UTR5 | SLC23A3(rna-XM_015290080.2:c.-494C>T) |
| 7:22262020 | 7 | 2.2E+07 | 3.87716E-12 | intronic | SLC23A3 |
| 7:22262211 | 7 | 2.2E+07 | 3.87716E-12 | exonic | SLC23A3 |
| 7:22262592 | 7 | 2.2E+07 | 3.87716E-12 | exonic | SLC23A3 |
| 7:22265567 | 7 | 2.2E+07 | 8.79644E-11 | intronic | SLC23A3 |
| 7:22266630 | 7 | 2.2E+07 | 2.51621E-09 | intronic | SLC23A3 |
| 7:22266822 | 7 | 2.2E+07 | 1.99061E-14 | UTR5 | NHEJ1(rna-XM_015290084.2:c.-305T>C,rna-XM_015290085.2:c.-305T>C,rna-XM_025152443.1:c.-305T>C) |
| 7:22266904 | 7 | 2.2E+07 | 8.63012E-11 | UTR5 | NHEJ1(rna-XM_015290084.2:c.-223C>T,rna-XM_015290085.2:c.-223C>T,rna-XM_025152443.1:c.-223C>T) |
| 7:22266951 | 7 | 2.2E+07 | 4.37592E-11 | UTR5 | NHEJ1(rna-XM_015290084.2:c.-176G>A,rna-XM_015290085.2:c.-176G>A,rna-XM_025152443.1:c.-176G>A) |
| 7:22266955 | 7 | 2.2E+07 | 4.37592E-11 | UTR5 | NHEJ1(rna-XM_015290084.2:c.-172T>C,rna-XM_015290085.2:c.-172T>C,rna-XM_025152443.1:c.-172T>C) |
| 7:22267260 | 7 | 2.2E+07 | 5.99561E-14 | exonic | NHEJ1 |
| 7:22267478 | 7 | 2.2E+07 | 5.99561E-14 | intronic | NHEJ1 |
| 7:22270780 | 7 | 2.2E+07 | 4.70996E-10 | intronic | NHEJ1 |
| 7:22272922 | 7 | 2.2E+07 | 1.6663E-08 | intronic | NHEJ1 |
| 7:22272977 | 7 | 2.2E+07 | 3.50985E-09 | intronic | NHEJ1 |
| 7:22281834 | 7 | 2.2E+07 | 1.33832E-14 | intronic | NHEJ1 |
| 7:22281861 | 7 | 2.2E+07 | 1.33832E-14 | intronic | NHEJ1 |
| 7:22281903 | 7 | 2.2E+07 | 1.33832E-14 | intronic | NHEJ1 |
| 7:22282120 | 7 | 2.2E+07 | 1.33832E-14 | intronic | NHEJ1 |
| 7:22282160 | 7 | 2.2E+07 | 7.37343E-09 | intronic | NHEJ1 |
| 7:22283044 | 7 | 2.2E+07 | 1.33832E-14 | intronic | NHEJ1 |
| 7:22283236 | 7 | 2.2E+07 | 1.33832E-14 | intronic | NHEJ1 |
| 7:22283615 | 7 | 2.2E+07 | 4.56711E-11 | intronic | NHEJ1 |
| 7:22283800 | 7 | 2.2E+07 | 5.02381E-14 | intronic | NHEJ1 |
| 7:22283906 | 7 | 2.2E+07 | 2.09677E-17 | intronic | NHEJ1 |
| 7:22284639 | 7 | 2.2E+07 | 1.11718E-09 | intronic | NHEJ1 |
| 7:22285643 | 7 | 2.2E+07 | 2.85186E-09 | intronic | NHEJ1 |
| 7:22285800 | 7 | 2.2E+07 | 7.55948E-10 | intronic | NHEJ1 |
| 7:22286923 | 7 | 2.2E+07 | 5.20595E-10 | intronic | NHEJ1 |
| 7:22287285 | 7 | 2.2E+07 | 8.52672E-10 | intronic | NHEJ1 |
| 7:22313372 | 7 | 2.2E+07 | 5.51522E-22 | intergenic | IHH(dist=3738),gene-MNR2(dist=2796) |
| 7:22313791 | 7 | 2.2E+07 | 5.51522E-22 | intergenic | IHH(dist=4157),gene-MNR2(dist=2377) |
| 7:22313809 | 7 | 2.2E+07 | 5.51522E-22 | intergenic | IHH(dist=4175),gene-MNR2(dist=2359) |
| 7:22313848 | 7 | 2.2E+07 | 5.51522E-22 | intergenic | IHH(dist=4214),gene-MNR2(dist=2320) |
| 7:22314077 | 7 | 2.2E+07 | 5.51522E-22 | intergenic | IHH(dist=4443),gene-MNR2(dist=2091) |
| 7:22314230 | 7 | 2.2E+07 | 5.51522E-22 | upstream | MNR2(dist=1938) |
| 7:22314246 | 7 | 2.2E+07 | 1.07565E-09 | upstream | MNR2(dist=1922) |
| 7:22314563 | 7 | 2.2E+07 | 5.51522E-22 | upstream | MNR2(dist=1605) |
| 7:22315182 | 7 | 2.2E+07 | 5.51522E-22 | upstream | MNR2(dist=986) |
| 7:22315618 | 7 | 2.2E+07 | 5.51522E-22 | upstream | MNR2(dist=550) |
| 7:22317198 | 7 | 2.2E+07 | 3.25382E-16 | intronic | MNR2 |
| 7:22317563 | 7 | 2.2E+07 | 2.51583E-09 | intronic | MNR2 |
| 7:22317877 | 7 | 2.2E+07 | 4.25636E-18 | intronic | MNR2 |
| 7:22318599 | 7 | 2.2E+07 | 5.51522E-22 | intronic | MNR2 |
| 7:22318769 | 7 | 2.2E+07 | 2.33676E-08 | intronic | MNR2 |
| 7:22318773 | 7 | 2.2E+07 | 2.33676E-08 | intronic | MNR2 |
| 7:22318804 | 7 | 2.2E+07 | 3.85493E-17 | intronic | MNR2 |
| 7:22318907 | 7 | 2.2E+07 | 5.51522E-22 | intronic | MNR2 |
| 7:22318910 | 7 | 2.2E+07 | 2.22168E-10 | intronic | MNR2 |
| 7:22319608 | 7 | 2.2E+07 | 4.83442E-12 | intronic | MNR2 |
| 7:22320748 | 7 | 2.2E+07 | 4.25636E-18 | intronic | MNR2 |
| 7:22321345 | 7 | 2.2E+07 | 4.25636E-18 | intronic | MNR2 |
| 7:22321362 | 7 | 2.2E+07 | 1.55885E-13 | intronic | MNR2 |
| 7:22321653 | 7 | 2.2E+07 | 2.33471E-11 | exonic | MNR2 |
| 7:22321805 | 7 | 2.2E+07 | 2.18136E-13 | exonic | MNR2 |
| 7:22323473 | 7 | 2.2E+07 | 6.19786E-09 | downstream | MNR2(dist=1375) |
| 7:22323522 | 7 | 2.2E+07 | 6.19786E-09 | downstream | MNR2(dist=1424) |
| 7:22354188 | 7 | 2.2E+07 | 4.7038E-09 | upstream | FEV |
| 7:22357986 | 7 | 2.2E+07 | 2.43116E-10 | intronic | FEV |
| 7:22358075 | 7 | 2.2E+07 | 2.43116E-10 | intronic | FEV |
|  |  |  |  |  |  |
|  |  |  |  |  |  |
| **GWAS significant InDels site annotation results** | | | | | |
| rs | chr | ps | p_wald | type | annovar gene |
| 7:22252639 | 7 | 2.2E+07 | 9.07421E-11 | Intronic | FAM134A |
| 7:22254877 | 7 | 2.2E+07 | 2.33645E-13 | Intronic | CNPPD1 |
| 7:22319063 | 7 | 2.2E+07 | 6.41326E-10 | Intergenic | IHH, MNR2 |
| 7:22319066 | 7 | 2.2E+07 | 7.27058E-22 | Intergenic | IHH, MNR2 |
| 7:22332732 | 7 | 2.2E+07 | 7.02237E-09 | Intronic | MNR2 |
| 7:22348431 | 7 | 2.2E+07 | 1.41551E-08 | Intronic | MNR2 |
