# Supplementary figures and images for: A heterozygous deletion and inversion at the NHEJ1‑IHH locus associated with shank length in Yunlong short-leg chicken
Source: BMC Genomics. 2026 May 18;27:607. doi: 10.1186/s12864-026-12943-0 (PMC13352712; doi:10.1186/s12864-026-12943-0)

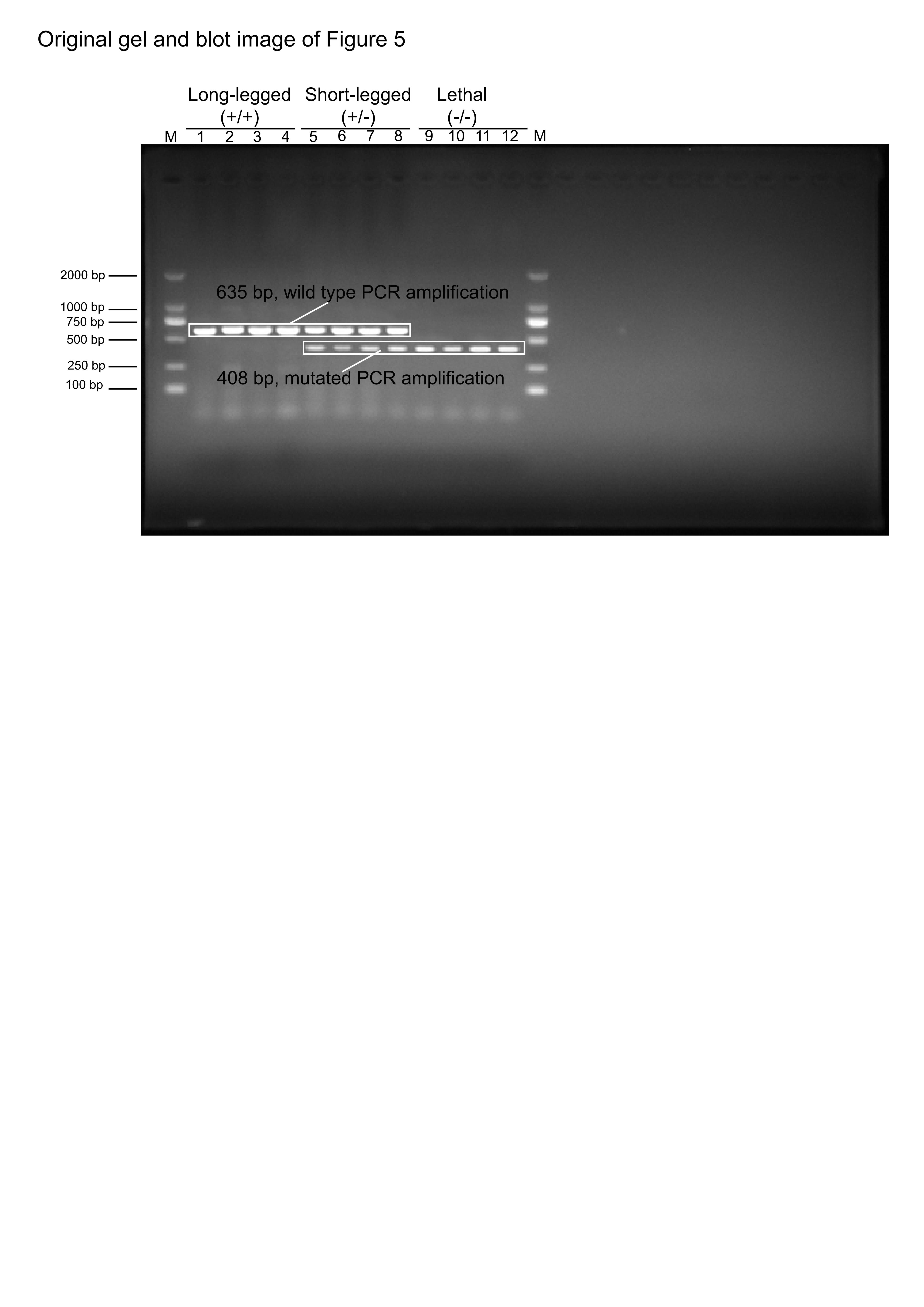

Supplement: Supplementary file 1 — Supplementary Material 1. [file 12864_2026_12943_MOESM1_ESM.zip › Original gel and blot image of Figure 5_01.jpg]

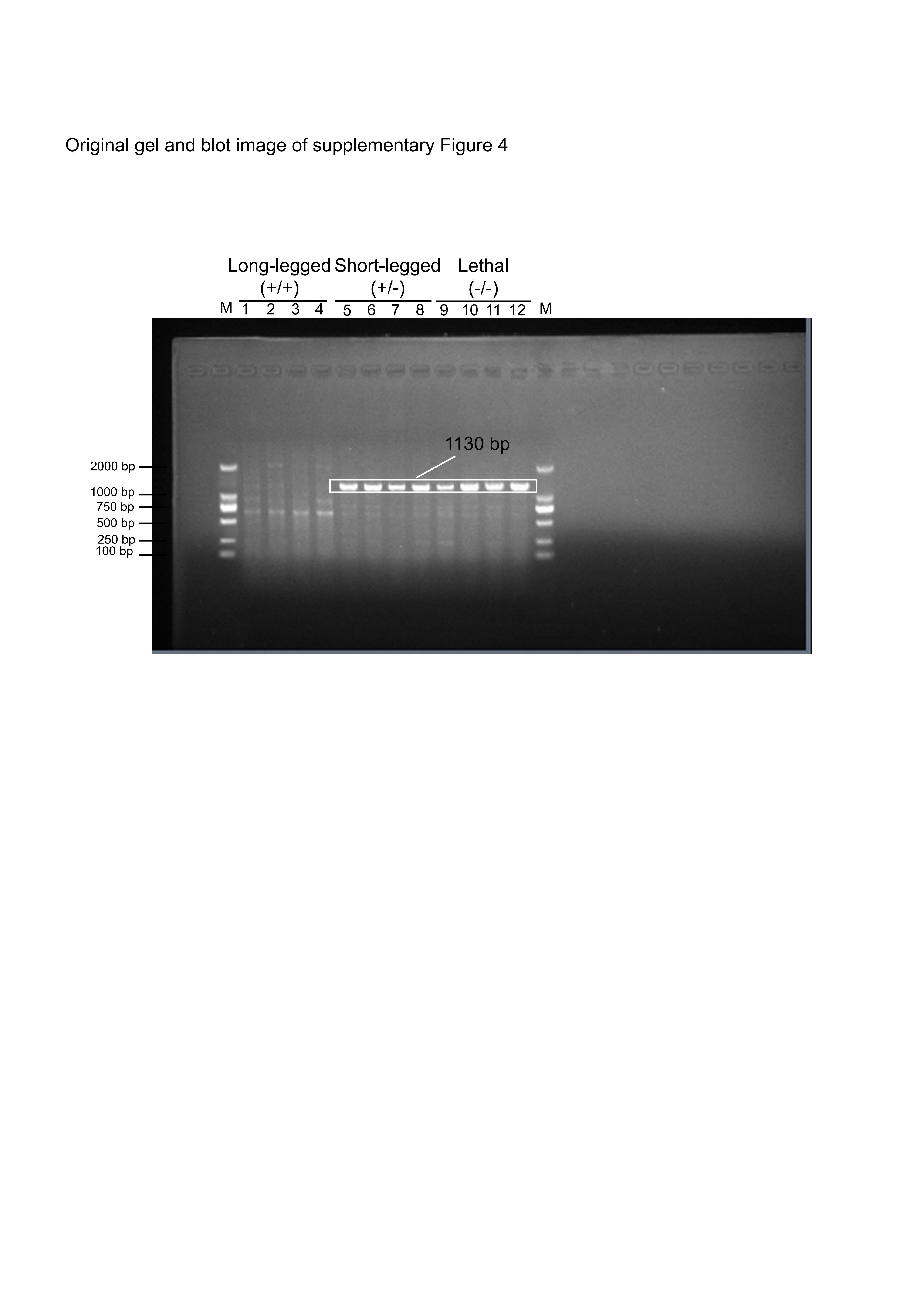

Supplement: Supplementary file 1 — Supplementary Material 1. [file 12864_2026_12943_MOESM1_ESM.zip › Original gel and blot image of supplementary Figure 4_01.jpg]
